# DHX29 functions as an RNA co-sensor for MDA5-mediated EMCV-specific antiviral immunity

**DOI:** 10.1371/journal.ppat.1006886

**Published:** 2018-02-20

**Authors:** Qingyuan Zhu, Peng Tan, Yinyin Li, Meng Lin, Chaoran Li, Jingrong Mao, Jun Cui, Wei Zhao, Helen Y. Wang, Rong-Fu Wang

**Affiliations:** 1 Center for Inflammation and Epigenetics, Houston Methodist Research Institute, Houston, TX, United States of America; 2 Institute of Biosciences and Technology, College of Medicine, Texas A&M University, Houston, United States of America; 3 Key Laboratory of Gene Engineering of the Ministry of Education and State Key Laboratory of Biocontrol, College of Life Sciences, Sun Yat-sen University, Guangzhou, China; 4 Xiangya Hospital, Central South University, Changsha, China; 5 Key Laboratory for Stem Cells and Tissue Engineering of the Ministry of Education, Zhongshan School of Medicine, Sun Yat-sen University, Guangzhou, China; 6 Department of Microbiology and Immunology, Weill Cornell Medicine, Cornell University, New York, NY, United States of America; University of Tennessee Health Science Center, UNITED STATES

## Abstract

Melanoma differentiation-associated gene-5 (MDA5) recognizes distinct subsets of viruses including Encephalomyocarditis virus (EMCV) of picornavirus family, but the molecular mechanisms underlying the specificity of the viral recognition of MDA5 in immune cells remain obscure. DHX29 is an RNA helicase required for the translation of 5’ structured mRNA of host and many picornaviruses (such as EMCV). We identify that DXH29 as a key RNA co-sensor, plays a significant role for specific recognition and triggering anti-EMCV immunity. We have observed that DHX29 regulates MDA5-, but not RIG-I-, mediated type I interferon signaling by preferentially interacting with structured RNAs and specifically with MDA5 for enhancing MDA5-dsRNA binding affinity. Overall, our results identify a critical role for DHX29 in innate immune response and provide molecular insights into the mechanisms by which DHX29 recognizes 5’ structured EMCV RNA and interacts with MDA5 for potent type I interferon signaling and antiviral immunity.

## Author summary

Cytosolic sensor melanoma differentiation-associated gene-5 (MDA5) specifically detects long-duplex RNAs in the genome of double-stranded (ds)RNA viruses such as Encephalomyocarditis virus (EMCV) whereas retinoic acid-inducible gene-I (RIG-I) preferentially recognizes vesicular stomatitis virus (VSV) to trigger signaling cascades that lead to production of interferons and cytokines. However, weak RNA binding capacity of MDA5 significantly attenuates the antiviral response. Here, we reveal that DHX29 as a co-sensor of MDA5 ensures the specific and efficient MDA5-RNA interactions leading to a complete MDA5-mediated antiviral signaling. Depletion of DHX29 substantially reduces the activity of MDA5 whereas activity of RIG-I remains intact. These findings provide a mechanism for DHX29 coactivation of MDA5 and a biological context for MDA5-RNA filaments in antiviral response.

## Introduction

RIG-I-like receptors, Toll-like receptors (TLRs), Nod-like receptors, and DNA sensors are germline-encoded pattern-recognition receptors (PRRs) that serve as the first line of defense against invading pathogens, including bacteria and viruses [1, 2]. PRRs possess the abilities to recognize pathogen-associated molecular patterns (PAMPS) and subsequent to the detection of PAMPs, PRRs trigger the activation of common type I interferon (IFN), NF-κB, inflammasome, and autophagy signaling pathways [1–5]. The consequent activation of these pathways induces the expression of cytokines and chemokines that facilitate the host pathogen defense and thus initiate the adaptive immune response. Among the PRRs, two RNA helicases such as Retinoic acid-inducible gene 1 (RIG-I, DDX58) and melanoma differentiation-associated protein 5 (MDA5), belonging to RIG-I-like receptor family, are considered to be the most important cytosolic viral RNA sensors [6]. Although both RIG-I and MDA5 can respond to West Nile virus and dengue virus [7], they also recognize specific sets of viruses [8]. While MDA5 primarily senses positive-strand viruses from the Picornaviridae family, such as Encephalomyocarditis virus (EMCV), RIG-I recognizes RNA viruses, including Sendai virus (SeV), vesicular stomatitis virus (VSV), and influenza virus [7, 9, 10]. Further studies and structural analysis of RIG-I and MDA5 showed that RIG-I recognizes the 5´-triphosphate group (5´-ppp) and blunt end of short (low molecular weight) RNAs with high affinity [11–14]. In contrast, MDA5 recognizes the internal duplex structure of long (high molecular weight) double strand (ds)RNAs with a weaker affinity [1, 15], thus resulting in the aggregation of MDA5 and its interaction with MAVS (VISA, IPS-1, Cardif) to trigger downstream type I IFN signaling cascade [16–19]. Of note, previous observation demonstrates that higher-order structure RNA comprising of both dsRNA and single strand RNA from EMCV-infected cells contain MDA5-stimulating activity [20], suggesting that other protein factors are involved in the selection and recognition of EMCV viral RNA. To this end, such potential candidate may be LGP2 (Laboratory of Genetics and Physiology 2), a cytoplasmic DExH helicase that shares the domain structure of RIG-I and MDA-5 with the exception of the CARD domains [1]. Paradoxically, LGP2 has been reported to exert both positive and negative effects on RIG-I and MDA5 regulation in different cell types in response to different viruses [15, 21–24], thus questioning its’ specificity to MDA5-mediated viral RNA selection and recognition. Based on these premises, the identification of protein factors that are involved in the selection and recognition of MDA5 for viral RNA is warranted.

In this study, our investigation directed towards the identification of factors required for recognition of MDA5 for viral mRNA revealed that the RNA helicase, DHX29 functions as a dsRNA co-sensor and exclusively interacts with MDA5 for enhanced RNA recognition in response to high molecular weight polyinosinic-polycytidylic acid [HMW-Poly(I:C)] treatment or EMCV infection in immune cells. In this respect, it is worth-mentioning that RNA helicases play critical roles in multiple biological processes, including protein translation initiation and stress granule formation [25–27]. In addition to the potential involvement in innate immune recognition and antiviral immunity [28, 29], several RNA helicases have also been identified as key factors in protein translation initiation [25, 30]. Recently, the RNA helicase DHX29 has been reported to be required for the formation and scanning of 43S translation initiation complexes on mRNAs with 5´-structured untranslated regions (UTRs) [31–33]. More importantly, mRNAs with internal ribosomal entry sites (IRES) occur in many Picornaviridae family viruses, including EMCV, and require DHX29 during translation initiation [34, 35]. Despite the important role of DHX29 in translation initiation of mRNAs with 5´ structured UTRs, particularly those from picornaviruses, its’ role in innate immune response to virus remains largely unknown. Interestingly, our mechanistic studies identify a previously unrecognized role of DHX29 in MDA5-mediated EMCV-specific type I IFN signaling and provide insights on the molecular mechanism by which DHX29 recognizes dsRNA and specifically interacts with MDA5 to render enhanced antiviral immunity.

## Results

### DHX29 positively regulates intracellular HMW Poly(I:C)-induced type I IFN signaling

**Fig 1:**
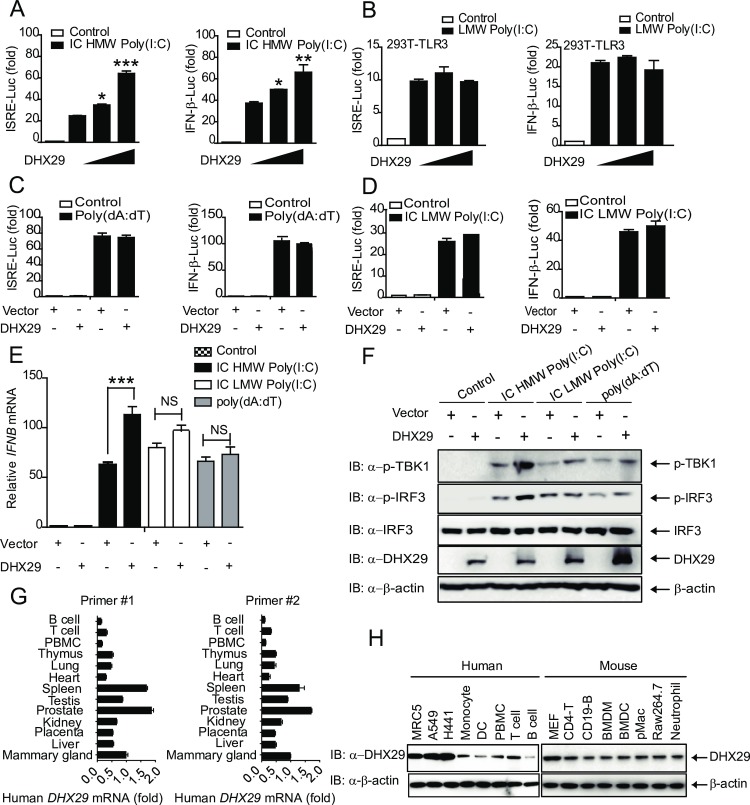
DHX29 positively regulates intracellular HMW Poly(I:C)-induced type I IFN response. (**A**) 293T cells and (**B**) 293T-TLR3 cells were cotransfected with IFN-β or ISRE promoter luciferase reporter (100 ng), Renilla luciferase internal control (pRL-TK), and empty vector or increasing concentrations of pcDNA3.1-HA-DHX29 (0, 50, and 100 ng). Transfected 293T and 293T-TLR3 cells were stimulated with intracellular (IC) HMW Poly(I:C) (5 μg/ml) and LMW Poly(I:C) (10 μg/ml), respectively. Fold changes in IFN-β-luciferase (Luc) and ISRE-Luc activities were determined. (**C**) IFN-β-Luc and ISRE-Luc activities in 293T cells stimulated with Poly(dA:dT) (5 μg/ml) (**D**) IFN-β-Luc and ISRE-Luc activities in 293T cells stimulated with IC LMW Poly(I:C) (5 μg/ml). (**E and F**) Real-time PCR of *IFN-*β *(IFNB)* mRNA (**E**) and immunoblot analysis (**F**) of TBK1 and IRF3 phosphorylation in empty vector- and pcDNA3.1-Flag-DHX29 (100 ng)-transfected 293T cells treated with IC HMW Poly(I:C), IC LMW Poly(I:C), or Poly(dA:dT). (**G**) Real-time PCR analysis of *DHX29 mRNA* expression in different human tissues by two different primer sets (Primer set #1 and Primer set #2). (**H**) Cell lysates (30 ug of total protein) of human lung cell lines MRC5, A549, H441, monocyte, dendritic cell (DC), T cell, B cell, mouse MEF, CD4-T cells, CD19-positive B cells, BMDM, BMDC, peritoneal macrophage (pMac), RAW 264.7, neutrophil were loaded on SDS-PAGE for western blotting analysis using anti-DHX29 and anti-β-actin antibodies. Data from (**A-E**) are plotted as the mean ± s.d. Results are representative of three independent experiments. *P < 0.05, **P <0.01, ***P <0.001 vs. the corresponding control. NS, not significant.

Despite the identification of many positive and negative regulators that control RIG-I function [36, 37], the knowledge on the functional regulation of MDA5 is scanty. In order to identify proteins involved in MDA5 regulation, we performed a functional screening of a cDNA expression sub-library for their ability to inhibit or enhance type I IFN signaling [38–42]. For this purpose, human embryonic kidney (HEK) 293T cells (293T cells) were cotransfected with the IFN-β promoter luciferase reporter, Renilla luciferase internal control (pRL-TK), and cDNA expression plasmids followed by stimulation with intracellular HMW Poly(I:C), low molecular weight (LMW) Poly(I:C), or intracellular DNA duplex Poly(dA:dT). Among them, DHX29 was identified as a positive regulator of intracellular HMW Poly(I:C)-induced IFN-β-Luc reporter activity as well as interferon-stimulated response element (ISRE)-luciferase (Luc) reporter activity, which only requires interferon regulatory factor 3 (IRF3) activation (Fig 1A). In contrast, DHX29 failed to increase ISRE-Luc and IFN-β-Luc activities in TLR3-expressing 293T cells (293T-TLR3) stimulated with LMW Poly(I:C) or 293T cells stimulated with Poly(dA:dT) or intracellular LMW Poly(I:C) (Fig 1B–1D). Consistent with these results, the ectopic expression of DHX29 markedly increased *IFN-*β transcription as well as TBK1 and IRF3 phosphorylation in response to intracellular HMW Poly(I:C), but not to intracellular LMW Poly(I:C) or Poly(dA:dT) treatment (Fig 1E and 1F*)*. Similar increase in *IFIT1* (encodes ISG56), *IFIT2* (encodes ISG54), and *CCL5* mRNA expression, was observed in response to intracellular HMW Poly(I:C) in DHX29 overexpressed cells (S1A–S1C Fig). Taken together, these results strongly suggest that DHX29 specifically enhances MDA5, but not TLR3 or RIG-I, mediated type I IFN signaling pathway.

DHX29 is expressed in human tissues and immune cells, especially high in prostate, spleen, mammary gland, testis, kidney and thymus using two sets of primers (#1 and #2) (Fig 1G). In addition to high expression of DHX29 in human lung cells [43], we showed that DHX29 protein could be detected in human peripheral blood mononuclear cells (PBMCs), monocyte, B cells, T cells, dendritic cells as well as in mouse T cells, B cells, bone marrow derived dendritic cells (BMDCs), peritoneal macrophages, bone marrow derived macrophages (BMDMs) and neutrophils (Fig 1H). Thus, our systematic investigation indicates that DHX29 is ubiquitously expressed in all tissues and cell types tested from human and mice, albeit with different expression levels, suggesting that DHX29 might have cell-type specific function in response to viral infection.

### DHX29 knockdown reduces HMW Poly(I:C)-induced type I IFN signaling in different cell types

We first assessed the effect of HMW Poly(I:C) stimulation on *DHX29* expression or induction, and found that *DHX29* expression was not affected by HMW Poly(I:C) or EMCV stimulation (S2A and S2B Fig). We next performed knockdown (KD) experiments and observed that three different *DHX29*-specific lentivirus short hairpin RNA (shRNA) constructs could effectively knockdown endogenous *DHX29* expression (S2C Fig). Furthermore, changes in cell viability were not observed for 3 days following transfection of shRNA targeting *DHX29* (S2D Fig).

**Fig 2:**
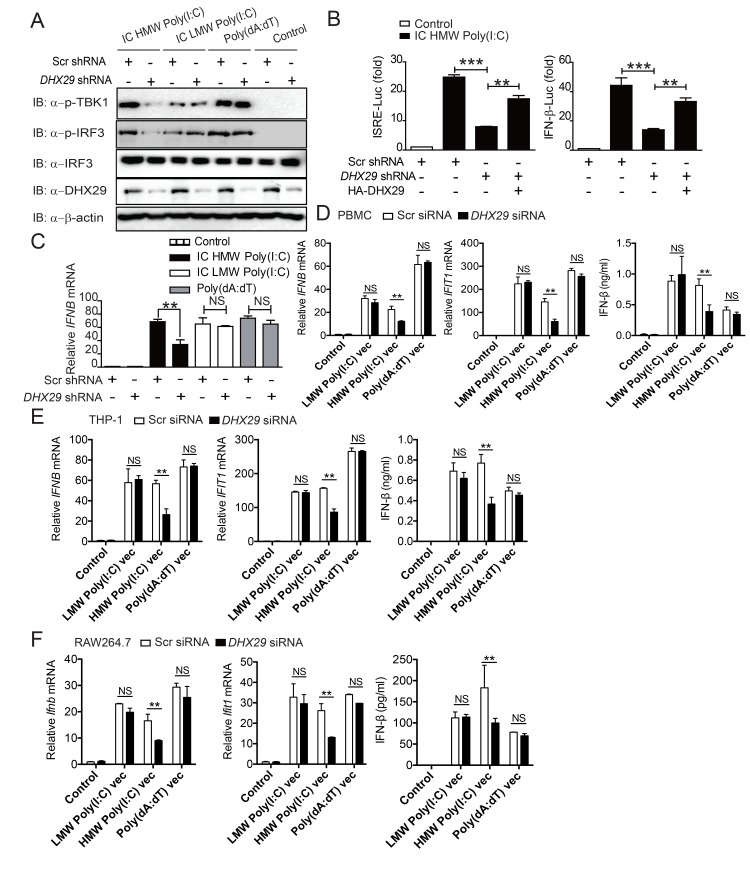
*DHX29* KD inhibits intracellular HMW Poly(I:C)-induced type I IFN response. (**A**) Immunoblot analysis of TBK1 and IRF3 phosphorylation in scrambled (Scr) shRNA- and *DHX29* shRNA-transfected 293T cells stimulated with intracellular (IC) HMW Poly(I:C), IC LMW Poly(I:C), or Poly(dA:dT). (**B**) ISRE- and IFN-β-luciferase (Luc) activities in Scr shRNA- and *DHX29* shRNA-transfected 293T cells stimulated with IC HMW Poly(I:C). ISRE-Luc activity was also determined in 293T cells cotransfected with HA-DHX29 (200 ng) and Scr shRNA or *DHX29* shRNA. IFN-β-Luc and ISRE-Luc activities were normalized to the Renilla luciferase internal control and presented as the fold increase relative to unstimulated control cells. (**C**) Real-time PCR analysis of *IFNB* mRNA expression in Scr shRNA- and *DHX29* shRNA-transfected 293T cells stimulated with IC HMW Poly(I:C), IC LMW Poly(I:C), or Poly(dA:dT). (**D-F**) PBMCs (**D**) and THP-1 (**E**) and RAW264.7 (**F**) cells were transfected with Scr siRNA or *DHX29* siRNA and stimulated with LMW Poly(I:C), HMW Poly(I:C) or Poly(dA:dT) in lyo/vec form (Vec). *IFNB*, *IFIT1*, and *IFIT2* mRNA expression levels and IFN-β production were determined using real-time PCR analysis and ELISA, respectively. Data from (**B-F**) are representative of three independent experiments and plotted as the mean ± s.d. *P < 0.05, **P <0.01, ***P <0.001 vs. the corresponding control.

*DHX29* KD markedly reduced TBK1 and IRF3 phosphorylation in response to intracellular HMW Poly(I:C) treatment, but not in response to LMW Poly(I:C) or Poly(dA:dT), in 293T cells (Fig 2A). Consistent with these results, *DHX29* KD greatly reduced ISRE-Luc and IFN-β-Luc activities in 293T cells stimulated with intracellular HMW Poly(I:C), which could be rescued by ectopic expression of HA-*DHX29* (Fig 2B). However, *DHX29* KD had little or no effect on ISRE-Luc and IFN-β-Luc activities in 293T-TLR3 cells stimulated with LMW Poly(I:C) or 293T cells stimulated with intracellular LMW Poly(I:C) or Poly(dA:dT) (S2E–S2G Fig). Furthermore, we found that the expression of several endogenous genes, including *IFN-*β, *IFIT1* and *IFIT2*, were reduced in cells transduced with *DHX29*-specific shRNAs in response to intracellular HMW Poly(I:C), but not intracellular LMW Poly(I:C) or poly(dAdT) (Fig 2C and S2H–S2J Fig), suggesting specific effects of DHX29 on HMW Poly(I:C)-induced type I IFN signaling.

To further confirm the role of DHX29 in the type I IFN signaling pathway, the effect of *DHX29* KD on the expression of type I IFN-stimulated genes and IFN-β production was determined in human and mouse immune cells. Human THP-1 cells, human peripheral blood mononuclear cells (PBMCs), and mouse RAW cells were transfected with *DHX29*-specific small interfering RNA (siRNA). High efficiency siRNA-mediated KD of *DHX29* was observed in all three types of cells (S3A Fig). Compared with the scrambled siRNA control, *DHX29* KD markedly reduced *IFN-*β, *IFIT1*, *IFIT2*, *and CCL5* mRNA expression and IFN-β production in PBMCs, THP-1 and RAW cells after treatment with LMW Poly(I:C) or HMW Poly(I:C) in lyo/vec form (Vec), but not to Poly(dA:dT) (Fig 2D–2F and S3B–S3E Fig). Taken together, our results suggest that DHX29 plays an important role in type I IFN signaling, and this function is conserved between human and mouse immune cells.

### Knockout of DHX29 in primary cells by CRISPR decreases type I IFN signaling in response to HMW Poly(I:C) and EMCV infection

**Fig 3:**
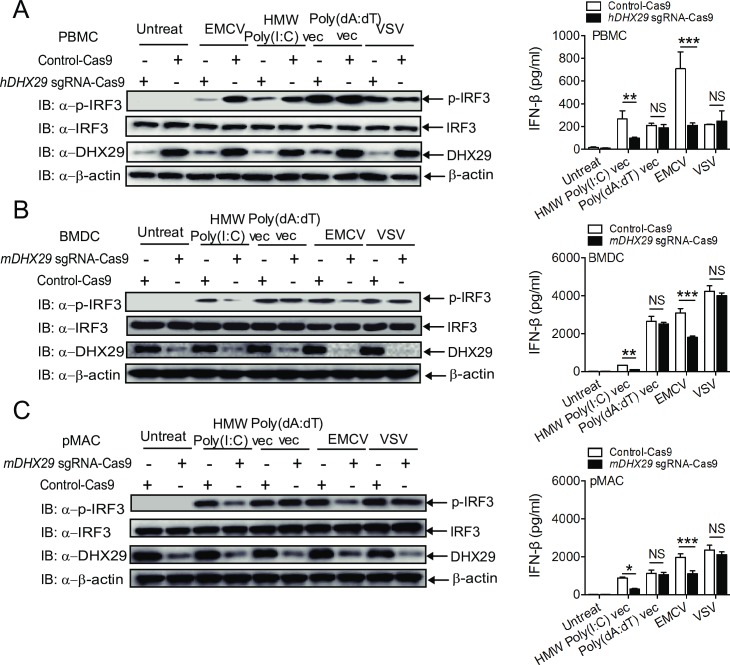
DHX29 knockout impairs MDA5 mediated type I IFN pathway in both human and mouse primary cells. (**A**) Human PBMCs, (**B**) murine BMDCs, and (**C**) murine peritoneal macrophages (pMAC) were transduced with control or mixed *DHX29* sgRNA/Cas9 lentiCRISPR viruses. Transduced cells were selected by puromycin treatment for 48 h to eliminate untransduced cells. Puromycin-resistant cells were cultured with fresh medium, left untreated or stimulated with HMW Poly(I:C) or Poly(dA:dT) in lyo/vec form (Vec) or infected with EMCV or VSV at an MOI of 10. Phosphorylation of IRF3 and IFN-β protein were determined by western blot and ELISA.

To further substantiate the biological role of DHX29 in type I IFN signaling in both human and mouse primary cells, we performed *DHX29* knockout (KO) experiments in human peripheral blood mononuclear cells (PBMCs), mouse bone marrow-derived dendritic cells (BMDCs) and peritoneal macrophages using a lentiviral-based Cas9-P2A-puromycin fusion CRISPR (*clustered regularly interspaced short palindromic repeats*). Of note, both human and murine *DHX29*-sgRNA/Cas9 lentiCRISPR could efficiently knock out DHX29 in puromycin-selected bulk PBMCs, BMDCs, and peritoneal macrophages, as determined by western blot and a surveyor assay (Fig 3A–3C and S4A–S4D Fig). *DHX29* KO markedly reduced TBK1 and IRF3 phosphorylation and IFN-β production in response to HMW Poly(I:C) in lyo/vec form (Vec) and EMCV, but did not affect IRF3 phosphorylation and IFN-β production in response to LMW Poly(I:C) Vec, Poly(dA:dT) or VSV in human PBMCs (Fig 3A and S4E Fig). Similar results were observed with *DHX29* KO by DHX29 sgRNA/Cas9 lentiCRISPR in murine BMDCs and peritoneal macrophages (Fig 3B and 3C and S4F and S4G Fig). Thus, these results suggest that *DHX29* KO markedly reduced type I IFN signaling in response to EMCV, but not VSV in immune cells, in contrast to what was observed in human airway epithelial cells [43]. It should be noted that all our KO experiments were performed on day 3–4 post transduction, since further long-term culture (after day 4) of DHX29 KO cells caused cell death (S4H Fig), which is consistent with previous observations [31–33, 44], showing that DHX29 is essentially required for protein translation, cell proliferation and survival. Collectively, our results gathered by deploying *DHX29* shRNA KD and *DHX29* KO approaches in human and murine primary immune cells, clearly suggest that DHX29 is required for type I IFN signaling in response to HMW Poly(I:C) treatment and EMCV infection.

### DHX29 is critically required for antiviral immunity against EMCV, but not to VSV infection

**Fig 4:**
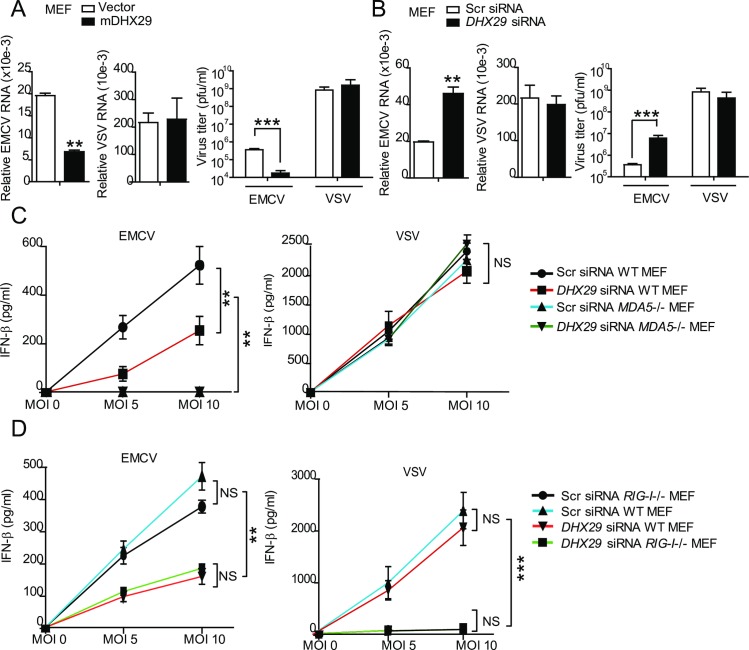
DHX29 is critically required for MDA5-mediated type IFN response to EMCV, but not VSV, infection. (**A**) DHX29 stably transfected (mDHX29) MEFs were infected with EMCV (multiplicity of infection [MOI] 5) or VSV (MOI 0.5). EMCV and VSV mRNA levels by real-time PCR analysis and virus titers were determined. Virus titers were expressed as plaque-forming units (pfu)/ml. (**B**) EMCV and VSV mRNA levels and titers were determined in scrambled (Scr) siRNA- and *DHX29* siRNA-transfected MEFs 24 h post-infection with EMCV (MOI 5) or VSV (MOI 1). (**C**) MDA5-knockout (*MDA5-/-*) and WT MEFs and (**D**) RIG-I-knockout (*RIG-I-/-*) and WT MEFS were transfected with *DHX29* siRNA or Scr siRNA and then infected with EMCV or VSV at the indicated MOIs. IFN-β production was determined by ELISA. Data from (**A-D**) are plotted as the mean ± s.d. and are representative of three independent experiments. *P < 0.05, **P <0.01, ***P <0.001 vs. the corresponding control. NS, not significant.

To further investigate the role of DHX29 in antiviral immune response and clarify the discrepancy between our data and those using human lung airway epithelial cells [43] on the specificity of antiviral immunity against EMCV and VSV, we examined whether the extent of DHX29 expression can modulate EMCV and VSV infection. The mRNA level and titer of EMCV, but not VSV, were significantly reduced in *DHX29* stably transfected (mDHX29) mouse embryonic fibroblasts (MEFs), when compared with empty vector-transduced MEFs (Fig 4A). Conversely, mRNA and titer of EMCV, but not VSV, were dramatically increased in *DHX29* siRNA-transfected MEFs in comparison to scrambled siRNA-transfected MEFs (Fig 4B). Our results suggest that the expression level of DHX29 can specifically modulate the infection ability of EMCV.

Because EMCV infection is known to activate MDA5-mediated type I IFN signaling, we further determined whether MDA5 is required for DHX29-mediated antiviral immunity. Our investigation with MDA5 wild-type (WT) and deficient MEFs demonstrated the lack of IFN-β secretion in *MDA5*-deficient MEFs in response to EMCV regardless of DHX29 status (Fig 4C). In contrast, *DHX29* KD in WT MEFs markedly decreased IFN-β release after EMCV infection, when compared with scrambled shRNA-transduced MEFs (Fig 4C). By contrast, we did not observe any difference in IFN-β production in *DHX29* KD or *MDA5*-deficient MEFs in response to VSV infection (Fig 4C). Overall, our single and double knock down studies strongly indicate that DHX29-mediated antiviral response to EMCV is dependent upon MDA5. Subsequently, we pursued to determine whether RIG-I is required for DHX29-mediated antiviral immunity. We performed similar experiments with WT and RIG-I-deficient MEFs, and found no difference in IFN-β production between scrambled siRNA transfected WT and scrambled siRNA-transfected *RIG-I*-deficient MEFs or between *DHX29*-specific siRNA-transfected WT and *DHX29*-specific siRNA transfected *RIG-I*-deficient MEFs in response to EMCV infection (Fig 4D). However, we observed marked reduction in IFN-β production in both *DHX29*-specific siRNA-transfected (WT and *RIG-I*-deficient) MEFs, compared with scrambled siRNA transfected (WT and *RIG-I*-deficient) MEFs after EMCV infection (Fig 4D), suggesting that *DHX29* KD, but not *RIG-I* deficiency, is responsible for the impairment of EMCV-induced IFN-β production. In contrast, there was no appreciable change in IFN-β production in response to VSV infection between *DHX29* siRNA- and scrambled siRNA-transfected WT MEFs (Fig 4D). As expected, RIG-I-deficient MEFs failed to produce IFN-β after VSV infection regardless of DHX29 status (Fig 4D). Taken together, these results clearly suggest that both DHX29 and MDA5 are specifically required for EMCV-, but not VSV-, induced antiviral immune response in MEFs.

### DHX29 enhances type I IFN signaling by binding to MDA5 after stimulation

**Fig 5:**
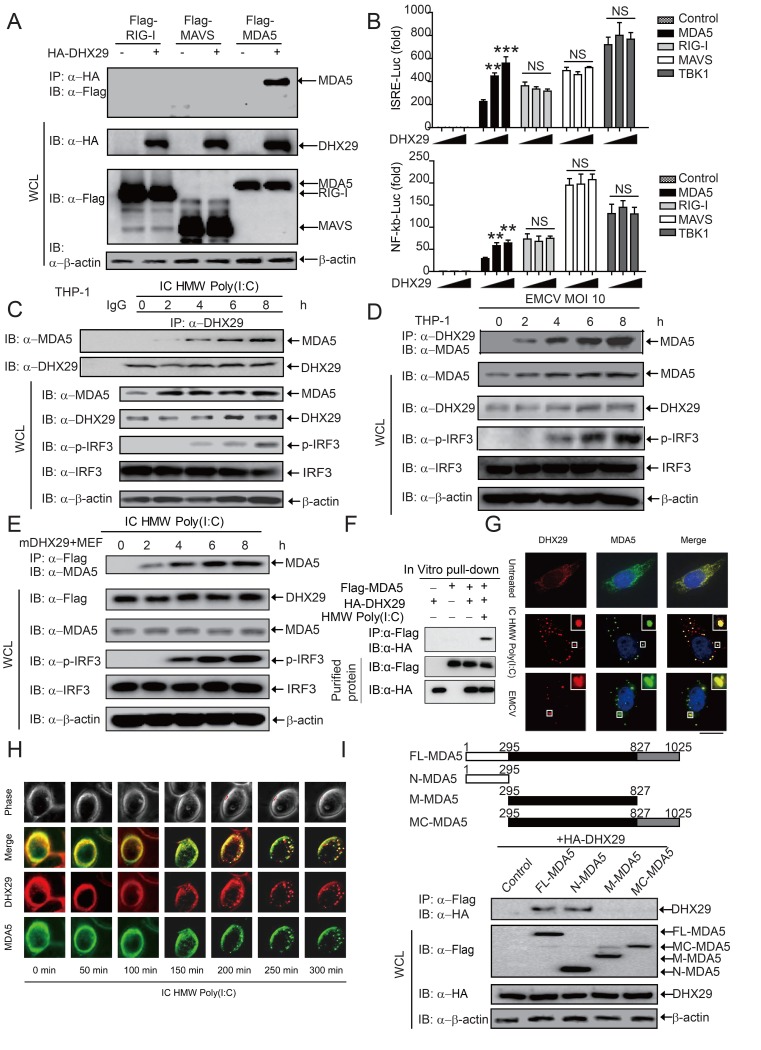
DHX29 directly interacts with MDA5 to enhance the type I IFN pathway. (**A**) Whole cell lysates (WCL) obtained from 293T cells cotransfected with *HA-DHX29* and *Flag-RIG-I*, *Flag-MDA5*, or *Flag-MAVS* plasmids were immunoprecipitated with anti-HA beads. The immunoprecipitated product was immunoblotted with anti-Flag antibody. (**B**) Luciferase activity of 293T cells transfected with an ISRE (upper panel) or NF-ĸB (bottom panel) luciferase reporter, together with vector for *RIG-I*, *MDA5*, *MAVS*, *TBK1*, along with empty vector (no wedge) or with increasing amounts (wedge) of expression vector for *DHX29*. (**C and D**) Coimmunoprecipitation assays were performed on WCL obtained from THP-1 cells infected with HMW Poly(I:C) (**C**) or EMCV (MOI 10) (**D**) at the indicated time points. Immunoprecipitation with anti-DHX29 antibody and immunoblotting with anti-MDA5, phosphorylated (p)-IRF3, and total IRF3 antibodies were performed. (**E**) *DHX29* stably transfected (mDHX29) MEFs were stimulated with HMW Poly(I:C) at the indicated time points. WCL were immunoprecipitated with anti-Flag beads and immunoblotted with anti-MDA5, p-IRF3, and total IRF3 antibodies. (**F**) Purified HA-DHX29 and Flag-MDA5 were co-incubated with or without HMW Poly(I:C) for 4 h. Pull-down experiments were performed with anti-Flag beads, followed by immunoblotting with anti-Flag or anti-HA antibody. (**G**) Confocal imaging analysis of the colocalization of DHX29 (red) and MDA5 (green) with or without HMW Poly(I:C) or EMCV stimulation for 5 h. Scale bar = 10 μm. (**H**) Time-course fluorescent microscopy analysis of the colocalization of DHX29 (red) and MDA (green) stimulated by HMW Poly(I:C) at indicated time points were recorded. Scale bar = 10 μm. (**I**) MDA5 deletion constructs were generated from full-length MDA5 (FL-MDA5) (*top panel*). WCL obtained from 293T cells cotransfected with *HA-DHX29* and *FL-MDA5*, *N-MDA5*, *M-MDA5*, or *C-MDA5* were immunoprecipitated with anti-Flag beads. The immunoprecipitated product was immunoblotted with anti-HA and anti-Flag antibodies. Data from (**B**) are plotted as the mean ± s.d. Results are representative of three independent experiments. *P < 0.05, **P <0.01, ***P <0.001 vs. the corresponding control. NS, not significant.

We next sought to determine whether DHX29 physically interacts with RNA sensors or adaptor molecules involved in the type I IFN signaling pathway. Accordingly, co-immunoprecipitation (CO-IP) assays were performed using 293T cells transfected with *HA*-*DHX29* and *Flag*-*RIG-I*, *Flag*-*MAVS*, or *Flag*-*MDA5*. Both human and mouse DHX29 interacted with MDA5, but not with RIG-I or MAVS (Fig 5A and S5A Fig). After demonstrating the specific interaction between DXH29 and MDA5, we investigated the effect of increasing amount of DHX29 expressing vector on the activation of MDA5.

293T cells were co-transfected with expression vectors encoding proteins RIG-I, MDA5, MAVS and TBK1 together with increasing amounts of expression vector that encodes DHX29 protein along with the ISRE or NF-ĸB luciferase reporter. We found that DHX29 specifically enhanced activation of the luciferase reporters induced by MDA5, but not others (Fig 5B). To further determine whether the endogenous interaction between DHX29 and MDA5 occur under physiological conditions, we treated THP-1 or mDHX29-expressing MEFs with intracellular HMW Poly(I:C) or EMCV infection. Immunoprecipitation and immunoblot analysis revealed no interaction between endogenous DHX29 and MDA5 in THP-1 cells stimulated with intracellular LMW Poly(I:C) (S5F Fig). The endogenous DHX29-MDA5 interaction was only observed following intracellular HMW Poly(I:C) treatment or EMCV infection (Fig 5C–5E). Consistently, DHX29 was detected in a complex with MDA5 in the presence of HMW Poly(I:C) by in vitro pull-down experiments using purified DHX29 and MDA5 proteins (Fig 5F). Colocalization of DHX29 and MDA5 after HMW Poly(I:C) or EMCV stimulation was detected by confocal imaging (Fig 5G). Specifically, the interaction of DHX29 with MDA5 was observed starting from 150 min after HMW Poly(I:C) stimulation analyzed by time-recording fluorescent microscopy (Fig 5H).

Interestingly, under stringent condition in the presence of our lysis buffer containing 1% Triton X-100, DHX29 did not interact with LGP2 or RIG-I, even after various kinds of stimulations. But in much lesser stringent condition such as in the presence of cell lysis buffer containing 0.1% NP-40 which was used in a recent report [43], we detected some weak association (S5G and S5H Fig). These results suggest that DHX29 specifically interacts with MDA5, but not with RIG-I or LGP2 protein, in a stringent condition which is optimal for determining the association in CO-IP assay. We further assessed the relative contribution of LGP2 and DHX29 to MDA5-mediated type I IFN signaling by knocking down or overexpression approaches. We found that KD of DHX29 markedly reduced IFN-β-luc activity induced by HMW Poly(I:C), which could be partially restored when LGP2 was overexpressed. Similarly, LGP2 KD strikingly reduced IFN-β-luc activity induced by HMW Poly(I:C), which could be partially restored when DHX29 was overexpressed (S5I Fig). Ectopic expression of both LGP2 and DHX29 further enhanced IFN-β-luc activity. Conversely, KD of LGP2 and DHX29 further markedly reduced IFN-β-luc activity (S5I Fig). These results suggest that LGP2 and DHX29 have additive and independent effects on MDA5-mediated type I IFN signaling pathway. Therefore, DHX29 enhances MDA5-mediated type I IFN signaling, independent of LGP2.

Additional experimental evidence document that the phosphorylation level of IRF3 induced by TBK1 is not affected by DHX29 in both WT and MAVS knockout cell (S5B Fig), All together the results obtained from luciferase reporter assay and interaction data in RIG-I knockout cell showed the similar pattern as observed in WT cell (Fig 5A and 5B, S5C and S5D Fig). Cumulatively, all these data suggest that DHX29 specifically functions in concert with MDA5 and acts upstream of MAVS. To determine whether the interaction between DHX29 and MDA5 is dependent upon ligand stimulation, we treated DHX29- and MDA5-expressing 293T cells with intracellular HMW Poly(I:C) and found that the interaction between DHX29 and MDA5 was induced following intracellular HMW Poly(I:C) treatment (S5E Fig). Furthermore, *DHX29* KD decreased the MDA5-MAVS interaction in response to HMW Poly(I:C) stimulation (S5J Fig). Henceforth, our mechanistic studies bolster us to suggest that DHX29 specifically binds to MDA5 and promotes the MDA5-MAVS interaction to enhance type I IFN signaling. To identify the interacting domains between MDA5 and DHX29, we generated different deletion constructs of MDA5, and tested for their abilities to interact with DHX29 (Fig 5I). DHX29 interacted with full-length MDA5 and the CARD domain-containing N-terminal fragment (N-MDA5), but not with M-MDA5 or MC-MDA5 (Fig 5I).

### DHX29 functions an RNA co-sensor with MDA5 for enhanced type I IFN signaling

**Fig 6:**
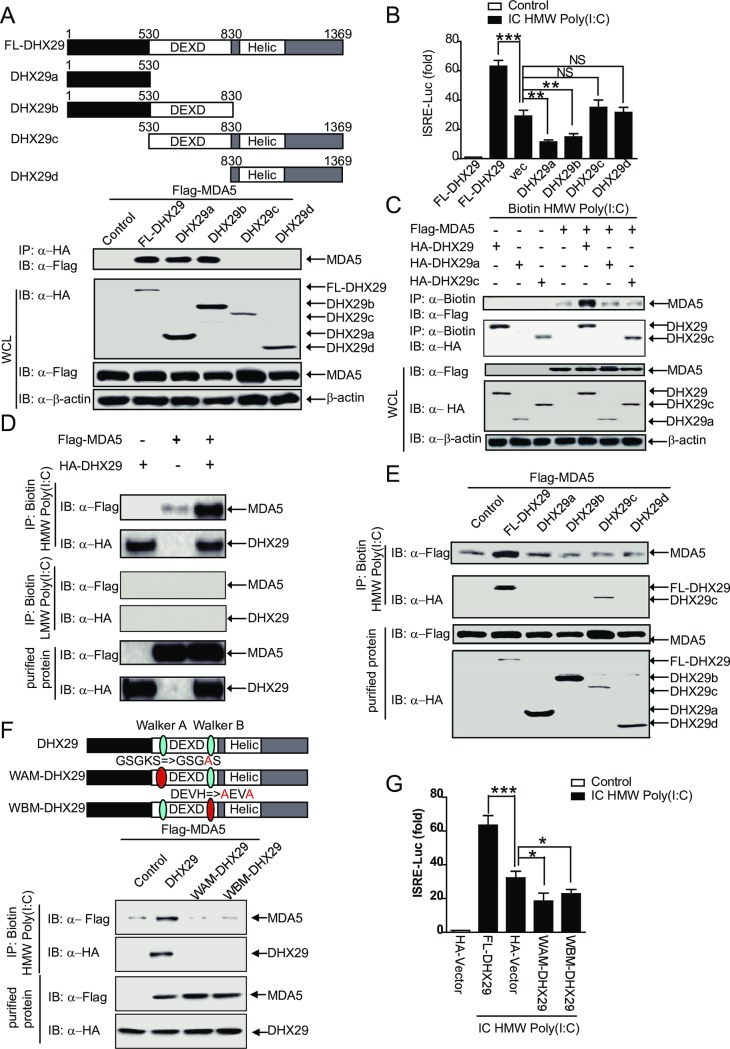
DHX29 functions an RNA co-sensor and interacts with MDA5 for enhanced RNA recognition and type I IFN signaling. (**A**) Schematic of DHX29 truncated forms (*top panel*). 293T cells were transfected with *Flag-MDA5* and full-length *HA-DHX29* (*FL-DHX29*), *HA-DHX29a*, *HA-DHX29b*, *HA-DHX29c*, or *HA-DHX29d* plasmids. Immunoprecipitation with anti-HA beads and immunoblotting with anti-HA and anti-Flag antibodies were performed (*bottom panel*). **(B**) 293T cells were cotransfected with ISRE-luciferase (Luc) and *FL-DHX29*, empty vector (vec), *DHX29a*, *DHX29b*, *DHX29c*, or *DHX29d* plasmids and stimulated with IC HMW Poly(I:C). ISRE-Luc activity was normalized to the Renilla luciferase internal control and presented as the fold increase relative to stimulated FL-DHX29 control cells. (**C**) Cell lysates were prepared from 293T cells transfected with full-length *HA-DHX29 or HA-DHX29a or HA-DHX29c* alone or in combination with *Flag-MDA5*, and then incubated with biotin-labeled HMW Poly(I:C) for 4 h. Immunoprecipitation was performed with anti-biotin beads, followed by immunoblotting with anti-Flag or anti-HA antibody. (**D**) Purified HA-DHX29 in the presence or absence of Flag-MDA5 was incubated with biotin-labeled LMW Poly(I:C) or HMW Poly(I:C) for 4 h. Pull-down experiments were performed with NeutrAvidin beads, followed by immunoblotting with anti-Flag or anti-HA antibody. (**E**) Purified Flag-MDA5 in the presence or absence of BSA (control), FL-DHX29, DHX29a, DHX29b, DHX29c, or DHX29d protein was incubated with HMW Poly(I:C) for 4 h. Pull-down experiments were performed with anti-biotin beads, followed by immunoblotting with anti-Flag or anti-HA antibody. (**F**) Schematic of DHX29 mutations in walker A (WAM-DHX29) and walker B (WBM-DHX29) sites. Purified Flag-MDA5 in the presence or absence of BSA (control), HA-DHX29, HA-WAM-DHX29, HA-WBM-DHX29 protein was incubated with HMW Poly(I:C) for 4 h. Immunoprecipitation with NeutrAvidin beads and immunoblotting with anti-Flag or anti-HA antibody were performed. **(G**) 293T cells co-transfected with ISRE-Luc and BSA (control), HA-DHX29, WAM-DHX29, or WBM-DHX29 were stimulated with IC HMW Poly(I:C). ISRE-Luc activity was normalized to the Renilla luciferase internal control and presented as the fold decrease relative to stimulated FL-DHX29 control cells. Data from (**B** and **G**) are representative of three independent experiments and plotted as the mean ± s.d. *P < 0.05, **P <0.01, ***P <0.001 vs. the corresponding control. NS, not significant.

To determine the specific role of DHX29 protein domains in the type I IFN pathway, four different DHX29 constructs (DHX29a [1–530 aa], DHX29b [1–830 aa], DHX29c [530–1369 aa], and DHX29d [830–1369 aa]) were generated and tested for their abilities to interact with MDA5 (Fig 6A). DHX29a and DHX29b, like full-length (FL) DHX29, were capable of binding to MDA5, whereas DHX29c and DHX29d failed to do so (Fig 6A), suggesting that the N-terminus (1–530 aa) of DHX29 is essential for DHX29 to interact with MDA5. In Fig 5I, DHX29 was shown to interact with N-MDA5. To confirm DHX29 interacts with MDA5 through N-DHX29 (DHX29a) and N-MDA5, we immunoprecipitated DHX29a with FL-MDA5 or deletions of MDA5, and found that DHX29a interacted only with FL- or N-MDA5 (S6A Fig). We next tested each of the four *DHX29* constructs for their abilities to enhance type I IFN signaling using the ISRE-Luc reporter. The *DHX29a* and *DHX29b* constructs did not increase but rather inhibited intracellular HMW Poly(I:C)- or MDA5-induced ISRE-Luc activity, whereas *DHX29c* and *DHX29d* had no effect on the ISRE-luc activity compared with the vector control (Fig 6B and S6B Fig), suggesting that full-length DHX29 is required for its ability to enhance MDA5-mediated type I IFN signaling.

To identify the molecular mechanisms by which DHX29 enhances MDA5-mediated type I IFN signaling, we tested whether DHX29 has the ability to bind to HMW Poly(I:C). To test this possibility, we performed a pull-down experiment with NeutrAvidin-beads from cell lysates of 293T cells cotransfected with biotin-labeled HMW Poly(I:C) along with MDA5, DHX29 fragments (FL, a or c), or MDA5 in combination with DHX29 fragments. We found that unlike DHX29a, FL-DHX29, DHX29c and MDA5 alone or in combination could be immunoprecipitated with anti-biotin beads (Fig 6C), suggesting that DHX29 functions as an RNA co-sensor to recognize HMW Poly(I:C). Furthermore, we performed similar experiments using LMW Poly(I:C) and found that only a small amount of DHX29 could be immunoprecipitated with anti-biotin beads (S6C Fig), suggesting that DHX29 preferentially binds to HMW Poly(I:C).

It has been known that MDA5 has a poor binding affinity for HMW Poly(I:C) and viral dsRNA [1, 15]. Therefore, we reasoned that DHX29 may enhance type I IFN signaling by recognizing the HMW Poly(I:C) and binding to MDA5. To test this possibility, we determined the ability of purified MDA5 and DHX29 to bind to HMW Poly(I:C). Indeed, we found that MDA5 interacted weakly to biotin-labeled HMW Poly(I:C), whereas DHX29 exhibited a strong HMW Poly(I:C)-binding ability, but not LMW Poly(I:C) (Fig 6D), suggesting that DHX29 is an RNA co-sensor. Importantly, co-incubation of MDA5 with DHX29 markedly enhanced the ability of MDA5 to bind to HMW Poly(I:C) (Fig 6D). To determine the DHX29 domain(s) required to enhance the HMW Poly(I:C)-binding capacity of MDA5, pull-down experiments were performed using purified full-length and truncated DHX29 proteins. Full-length DHX29, but not its truncated forms, enhanced the ability of MDA5 to bind to HMW Poly(I:C) (Fig 6E). Interestingly, full-length DHX29 and truncated DHX29c, but not DHX29a, DHX29b, or DHX29d, were pulled down with biotin-labeled HMW Poly(I:C) (Fig 6E), suggesting that DHX29c (containing the DEXD and helicase domains) has a strong HMW Poly(I:C)-binding ability. Taken together, these results suggest that DHX29 interacts with MDA5 through its N-terminus and binds to HMW Poly(I:C) through its DEXD and helicase domains, thus bringing MDA5 and HMW Poly(I:C) together and enhancing their interaction. These results may explain why full-length DHX29 is required to enhance the HMW Poly(I:C)-binding capacity of MDA5.

Because DHX29 contains two ATP binding motifs (Walker A and B) in the DEXD motif-containing domain [33], we next sought to determine whether these ATP binding motifs are necessary for DHX29 to sense HMW Poly(I:C) by introducing mutations in the Walker A and B sites of DHX29 (WAM-DHX29 and WBM-DHX29). Unlike full-length DHX29, WAM-DHX29 and WBM-DHX29 failed to bind to HMW Poly(I:C) or to increase the HMW Poly(I:C)-binding ability of MDA5 (Fig 6F). Consistent with these observations, we showed that like DHX29a and DHX29b, WAM-DHX29 and WBM-DHX29 did not increase but rather inhibited MDA5-mediated ISRE-Luc activity, as compared to full-length DHX29 (Fig 6G). The inhibitory effects of WAM-DHX29, WBM-DHX29, DHX29a, and DHX29b on HMW Poly(I:C)-induced ISRE-Luc activity were increased with increasing amounts of gene expression (S6D Fig), thus exerting a dominant negative effect on DHX29 function as an RNA sensor. Taken together, these results indicate that DHX29 functions as a sensor to recognize HMW Poly(I:C) and to interact with MDA5. The N-terminal domain of DHX29 is required for MDA5 binding, whereas the DEXD (Walker A and B)-containing domain is required for RNA binding. Thus, both the N-terminal and DEXD/helicase domains of DHX29 are required for its function as an RNA co-sensor to promote MDA5-mediated type I IFN signaling.

Dephosphorylation of MDA5 and RIG-I is a key step of their activation [37, 45, 46]. To test whether DHX29 is required for MDA5 activation, we used wildtype, non-interacting (DHX29c) and inactive (WAM-DHX29 and WBM-DHX29) form of DHX29 to test the phosphorylation level of MDA5 and RIG-I. However, we did not detect any significant change in phosphorylation of RIG-I and MDA5 with different forms of DHX29 in the presence or the absence of stimulation (S6E and S6F Fig).

### DHX29 forms the aggregation with MDA5 on RNA

**Fig 7:**
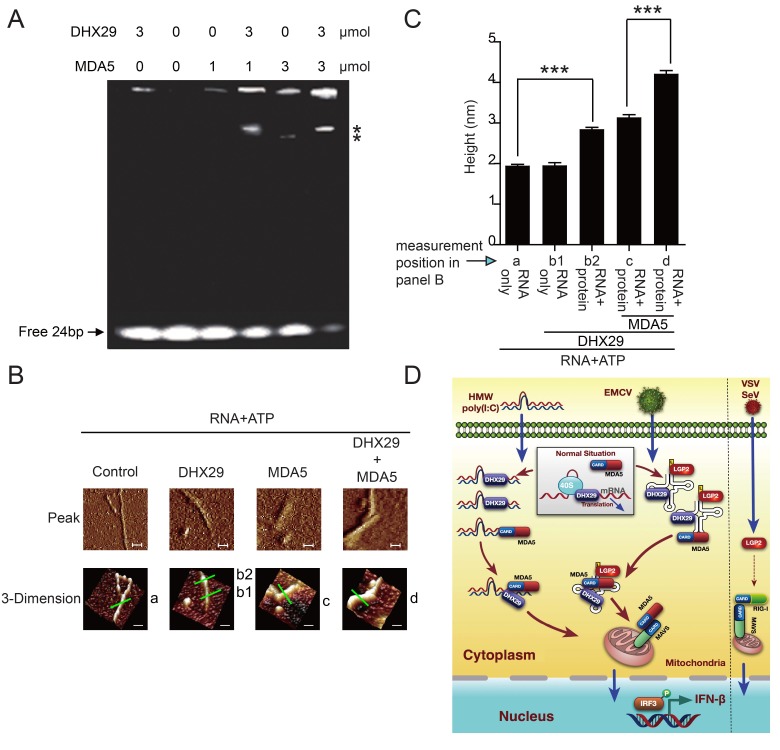
DHX29 forms the aggregation with MDA5 on RNA. (**A**) Electrophoretic mobility shift assay of purified MDA5 incubated with 24 bp RNA for 40 min on ice in the presence or absence of DHX29 protein. (**B**) AFM images of purified MDA5 protein in the presence and absence of DHX29 protein. (**C**) Section mode analysis was done to determine the height of the RNA-protein complexes. a, RNA alone (control); b1 and b2, RNA-DHX29; c, RNA-MDA5; and d, MDA5 plus DHX29. Data are plotted as the mean ± s.d. of ten independent site measurements. *P < 0.05, **P <0.01, ***P <0.001 vs. the corresponding control. (**D**) A proposed working model to illustrate the critical role of DHX29 in the control and activation of MDA5-mediated RNA recognition and type I IFN signaling.

Several recent studies show that MDA5 forms filaments upon binding to dsRNA, which is critical for the activation of the IFN-β signaling pathway [47, 48]. To determine whether DHX29 promotes the aggregation of MDA5 filaments along dsRNA, we performed electrophoretic mobility shift assays (EMSAs). Although DHX29 and MDA5 protein alone could bind to dsRNA oligonucleotides, dsRNA-binding activity in both mature (higher band) and intermediate forms (lower band) was enhanced when DHX29 and MDA5 were combined (Fig 7A). To further confirm the binding enhancement, we utilized atomic force microscopy (AFM) to image the RNA-protein complex. DHX29 was able to bind to Poly(I:C) but did not form filaments (Fig 7B). However, DHX29 promoted the aggregation of MDA5 filaments on Poly(I:C) dsRNA (Fig 7B). Because the mica plate flattens at the molecular level, the height (vertical distance) attribution could be used to represent the diameter of the RING structure of the protein-RNA complex (Fig 7C). We obtained 10 individual “section mode” analyses of the RNA-protein complexes. Compared with the 2 nm diameter of dsRNA alone (standard height for double strand HMW Poly[I:C]), the diameter of the dsRNA-MDA5-DHX29 complex was around 4 nm, and the diameter of both the dsRNA-MDA5 and dsRNA-DHX29 complexes was around 3 nm (Fig 7C). These results suggest that DHX29 interacts with dsRNA and MDA5 to form the aggregation of DHX29-MDA5 along dsRNA, by which DHX29 promotes MDA5 activation.

## Discussion

RIG-I and MDA5 are key RNA sensors that recognize different sets of viruses. MDA5 primarily senses positive-strand viruses from the Picornaviridae family, such as EMCV, whereas RIG-I recognizes SeV, VSV, and influenza virus [7, 9, 10]. Although it is not clear how RIG-I and MDA5 specifically respond to different viruses, it is likely that differences in viral RNA ligand specificity (5´ ppp end of short RNA for RIG-I vs. internal duplex structure of long dsRNA for MDA5) and RNA binding affinity (high affinity for RIG-I vs. low affinity for MDA5) may contribute to the distinct viral recognition of MDA5 and RIG-I in the innate immune response. Recently, several RNA helicases have been implicated in viral immunity and protein translation [25]. For example, DDX1, DDX3, DDX5, DDX6, DDX24, DDX56, DDX60, DHX9, and DHX30 are implicated with human immunodeficiency virus, hepatitis B virus, hepatitis C virus, herpes simplex virus, VSV replication and innate immune response [26, 49, 50]. In particular, DDX1, DDX21, and DHX36 form a complex with adapter protein, TIR-domain-containing adapter-inducing interferon-β (TRIF) to recognize dsRNA from influenza virus and reovirus [51]. DDX41 can recognize intracellular DNA and activate STING-mediated type I IFN signaling [52]. However, potential RNA helicase(s) involved in the recognition of EMCV remain to be identified. In this study, we have provided compelling evidence that the RNA helicase DHX29 functions as an RNA co-sensor along with MDA5, and plays a critical role in innate immune signaling and immunity in response to HMW Poly(I:C) treatment and EMCV infection, but definitely not to LMW Poly(I:C) treatment or VSV infection. However, in contrary to our observation, a previous study reported that DHX29 is expressed solely in lung airway cells, but not in human PBMCs (such as B cells and T cells), primary monocytes, THP-1 cells and 293T cells, and thus does not play a role in type I IFN signaling in immune cells [43]. Moreover, previous study suggested that DHX29 interacts with RIG-I and functions as a co-receptor for sensing poly I:C, poly dAdT:dAdT and VSV infection [43]. However, the interaction between DHX29 and MDA5 was never explored in their study [43].

To demonstrate the importance of DHX29 expression, several studies have shown that DHX29 is essentially required for protein translation, cell proliferation and survival [31–33, 44]. Consistent with these results, we show that KD or KO of DHX29 leads to cell death after 5 days in cell culture. More importantly, we demonstrate that both DHX29 and MDA5 function as RNA co-sensors and are required for the dsRNA-binding and type I IFN signaling in response to HMW poly I:C and EMCV, but not VSV infection. Our data on the specific recognition of EMCV by DHX29 are quite concordant with several recent studies showing the absolute requirement of DHX29 for the formation and scanning of 43S translation initiation complexes on mRNAs with 5´-structured UTRs, such as those from EMCV [31–33]. The translation of such mRNAs requires DHX29 in 43S translation initiation complexes to unwind structured mRNA and identify the correct initial start codon [34, 35]. These studies also suggest that DHX29 can recognize mRNAs with 5´-structured UTRs present in many Picornaviridae family viruses, including EMCV. Our results obtained with shRNA-mediated KD as well as CRISPR/Cas9-mediated KO of *DHX29* in various cell types, further substantially demonstrate the specificity and requirement of DHX29 as an RNA co-sensor in the innate immune response to HMW Poly(I:C) treatment and EMCV infection. Moreover, the presence of DXH29 in both human and mouse cells supports for its functional conservation between human and mouse. Hence, our results unequivocally establish that DHX29 is expressed in all types of immune cells tested, including PBMC, monocyte, B cells, T cells, macrophages and BMDC, and is essentially required for cell proliferation and survival; Importantly, our results have clearly demonstrated that DHX29 functions as an MDA5-specific RNA sensor in response to HMW Poly(I:C) and EMCV, but not to LMW Poly(I:C) or VSV, in immune cells tested. Further evidence shows that DHX29 alone cannot trigger type I IFN signaling in immune cells; it must interact with MDA5, but not RIG-I, to activate the type I IFN signaling pathway in various immune cell types, including THP-1, human PBMCs, murine macrophages and DCs. Of note, DHX29 does not interact with MDA5 in uninfected or untreated cells, but rather is induced after HMW Poly(I:C) treatment or EMCV infection. DHX29 does not interact with LGP2 or RIG-I even after viral infection, suggesting that the N-terminus of DHX29 specifically binds to the N-terminal (CARD-containing) domain of MDA5. However, unlike full-length DHX29, the truncated N-terminus of DHX29 (without the C-terminus) inhibits MDA5-mediated type I IFN signaling, suggesting that full-length DHX29 is required for its ability to enhance type I IFN signaling.

The role of LGP2 in antiviral immunity is controversial. Both positive and negative effects on RIG-I- and MDA5-mediated type I IFN signaling have been reported in response to different viruses [15, 21–24]. One study shows that LGP2 is not essential for the induction of innate immune response, but rather is required for controlling antigen-specific CD8-positive T cell survival in response to virus infection [53]. However, several studies suggest that LGP2 works upstream of RIG-I and MDA5 to potentiate viral RNA-induced signaling and is essential for RIG-I- and MDA5-mediated type I IFN signaling and antiviral immunity [21, 54]. In particular, LGP2 recognizes L region of EMCV genome and promotes the type-I interferon signal in response to EMCV infection [55]. Our results indicate that both LGP2 and DHX29 could markedly affect MDA5-mediated type I IFN response to HMW poly(I:C). Co-expression of LGP2 and DHX29 increases MDA5-mediated type I IFN signaling more than those obtained with either LGP2 or DHX29 expression alone. Ectopic expression of DHX29 can partially rescue HMW poly(I:C)-induced type I IFN signaling reduced by LGP2 KD. Thus, our study suggests that DHX29 positively regulate MDA5-mediated type I IFN signaling independent of LGP2. Consistent with this notion, we show that DHX29 does not interact with LGP2. Thus, our results identify a previously unrecognized role of DHX29 in MDA5-mediated type I IFN signaling and anti-EMCV immunity. Another MDA5 activator PACT was reported to promote MDA5 signaling by promoting MDA5 oligomerization [56], it will be of interest to investigate the relationship between DHX29 and PACT in the near future studies.

To understand the molecular mechanisms by which DHX29 enhances MDA5-mediated type I IFN signaling, we show that DHX29 has the ability to bind to HMW Poly(I:C) with a strong binding affinity compared with the low HMW Poly(I:C)-binding affinity of MDA5. This is in agreement with previous studies showing that MDA5 has a poor binding affinity for HMW Poly(I:C) and viral dsRNA [1, 15]. Coexpression of *DHX29* and *MDA5* markedly increases the binding capacity of MDA5 to HMW Poly(I:C), suggesting that DHX29 and MDA5 function as co-sensors for detecting EMCV infection. Further experiments demonstrate that the DEXD (containing Walker A and B ATP binding motifs) and helicase domains of DHX29 are responsible for HMW Poly(I:C) binding. Thus, it appears that DHX29 specifically interacts with MDA5 through its N-terminus and binds to HMW Poly(I:C) through its C-terminal DEXD and helicase domains, thus bringing MDA5 and HMW Poly(I:C) together. Indeed, our EMSA and AFM results show that the dsRNA-binding activity of DHX29 and MDA5 is much better than MDA5 alone. Overall, further analysis is needed to clarify whether the helicase activity of DHX29 is required for its function as co-sensor for MDA5-dsRNA. The potential role of DHX29 in the modulating of ATPase activity and oligomerization of MDA5 [57–59] requires further investigation, although our study strongly indicates that DHX29 bring HMW Poly(I:C) and MDA5 together for enhanced dsRNA binding capacity and consequent type I IFN signaling. Although DHX29 mainly functions as a core component of translation initiation complexes required for the translation of mRNA with 5´-structured UTR under normal physiological conditions, it may bring these structured RNAs in close proximity to MDA5 to promote MDA5-RNA binding and MDA5 aggregation along dsRNA after EMCV infection or HMW Poly(I:C) treatment, which in turn leads to the activation of MDA5 for consequent downstream type I IFN signaling.

Based on the results presented here and previously published data, we propose a working model to illustrate the role of DHX29 in MDA5-mediated innate immune response to HMW Poly(I:C) treatment and EMCV infection (Fig 7D). During normal physiological conditions, DHX29 is mainly involved in its function in the protein translation. However, DHX29 may switch its function to sense and initiate MDA5-mediated type I IFN signaling after EMCV infection or HMW Poly(I:C) treatment. By contrast, the major function of LGP2 is to regulate both RIG-I and MDA5 mediated innate immune signaling in response to different viral infection. Overall, our findings identify DHX29 as an RNA co-sensor with MDA5 in innate immune response and provide molecular insights into the mechanisms by which DHX29 controls EMCV-specific and MDA5-mediated antiviral immunity.

## Materials and methods

### Ethics statement

Buffy coats of blood from healthy donors (from the Gulf Coast Regional Blood Center, Houston, TX) were used for isolation of PBMCs (human peripheral blood mononuclear cells) by density-gradient centrifugation with Lymphoprep (Nycomed Pharm). All blood samples were anonymized. The use of PBMCs was in accordance with institutional guidelines on human cell research and the approved protocol (Protocol #IBC00000357) by the Institutional Review Board of Houston Methodist Research Institute. Animal experiments in this study were approved and carried out in accordance with protocol (Protocol #AUP-0115-0005) provided by the Institutional Animal Care and Use Committee (IACUC) at Houston Methodist Research Institute. IACUC uses the National Institute of Health (NIH) Guide for the Care and Use of Laboratory Animals, which is based on the U.S. Government Principles for Utilization and Care of Vertebrate Animals Used in Testing, Research, and Training.

### Reporter assay

Human embryonic kidneys (HEK) 293T (2x10^5^) cells (ATCC, CRL-3216) were seeded into 24-well plates the day before transfection. Cells were cotransfected with the IFN-β or ISRE promoter luciferase reporter and Renilla luciferase internal control (pRL-TK) using Lipofectamine 2000 (Invitrogen) as previously described [60]. Cells were stimulated with DHX29, RIG-I, or MDA5 ligand 24 h post-transfection. Empty pcDNA3.1 HA/Flag/Myc tagged vector was used to ensure equal amounts of DNA among the wells. Luciferase activity was determined using the Dual-Luciferase Assay (Promega) with the Luminoskan Ascent Luminometer (Thermo Scientific) as previously described [41]. Reporter gene activity was normalized to the internal control.

### Virus and viral infection assays

Encephalomyocarditis Virus (EMCV; ATCC, VR-1762) and Vesicular stomatitis virus (VSV, Indiana; ATCC, VR-1415) were amplified in Vero cells (African green monkey kidney cell line; ATCC, CCL-81) and 293T cells, respectively, and stored at -80°C until use. After three cycles of freeze-thawing to release intracellular virus, the supernatant was collected by spinning and used in plaque-forming assays.

### Construction of DHX29 sgRNA/Cas9 LentiCRISPR and viral transduction

We designed human and murine DHX29 sgRNA as previously described [61]. Four hDHX29 and 4 mDHX29 sgRNAs were designed and cloned into the BsmB1 site of lentiCRISPR vector containing Cas9-P2A-puromycin as previously described [61] and verified by sequencing analysis. The sgRNA-containing plasmids were transfected into 293T cells with pCMV-VSV-G plasmids and pΔ8.9. After two days, the virus-containing medium was subjected to ultracentrifugation (20000 x *g* at 4°C for 2 h) and frozen at -80°C. Murine Bone marrow-derived dendritic cells (BMDCs) were generated by flushing bone marrow cells from femurs and tibiae of mice and were cultured in RPMI media containing 10% FBS supplemented with GM-CSF and IL-4. Peritoneal macrophages (pMAC) were obtained by injecting mice with 4% (v/v) thioglycollate (Beckton Dickson), and peritoneal cavities were flushed after 3 days with RPMI media/2% FBS. Isolated PBMCs, BMDCs, and pMAC were transduced with 4 mixed DHX29-sgRNA-containing lentiCRISPR viruses, respectively. Transduced cells were selected in the presence of puromycin (Invivogen) for 48 h and subjected to ligand stimulation or viral infection.

### Immunofluorescence assay and confocal microscopy

The immunofluorescence assays and confocal microscopy were conducted as described previously [62]. 293T cells grown on glass-bottom dish (MatTek) in complete medium were co-transfected with fluorescently tagged expression vectors for DHX29 (DsRed2) and low concentration of MDA5 (GFP) for 12–16 hr. Cells were either left untreated or treated with LMW Poly(I:C) or EMCV for 5 h. Cells were live-recorded at every 50 min or fixed in 4% paraformaldehyde solution in PBS at room temperature for 15 min. The nucleus was then labeled with DAPI for 5 min in the dark and then followed by three washes in PBS. Samples were then visualized using Nikon Eclipse Ti-E microscope. All acquired images were analyzed and the correlation coefficient (r) of pixel intensity values was extracted by using the Nikon NIS-Elements AR package or the ImageJ (NIH) software.

### Poly(I:C) pull-down assay

For in-cell pull-down assays, clarified whole cell lysates (WCL) transfected with plasmid constructs expressing HA- or Flag-tagged protein were preincubated with NeutrAvidin beads for 2 h (Pierce). Biotin-labeled Poly (I:C) was added, and WCL were incubated for an additional 4 h at 4°C. For cell-free pull-down assays, WCL transfected with HA- or Flag-tagged protein were incubated with anti-HA or anti-Flag beads (Sigma), and proteins were eluted from the beads using the corresponding peptide. The purified proteins were then added to the NeutrAvidin beads and coincubated with Poly(I:C) for 4 h. After incubation, protein complexes were washed 5 times, boiled in 4X loading buffer, and subjected to SDS-PAGE.

### EMSA and AFM

24bp HPLC-grade RNA oligonucleotide 5´-GCGUCGUACGCUAG CGUACGACGC-3´ was purchased from Integrated DNA Technologies. Sample preparation was performed as previously described [48]. Samples were subjected to 4% native PAGE and stained with gel-red (Biotium). For AFM, we followed previously described protocol with modification[48]. Briefly, we mixed 10 ng HMW Poly(I:C) and 5 ng purified MDA5 protein in 10 μl of buffer and then incubated in the presence or absence of 10 ng DHX29 in buffer A (pH 7.4, 50 mM NaH2PO4, 50 mM glycine, 14 mM succinic acid, 10 mM MgCl2, 10 μM ZnCl2, 5 mM DTT and 2.5 mM ATP) for 40 min on ice. The mixtures were dropped onto the flat mica plate (molecular level) for 10 min and then washed 5 times with PBS to remove excess salt. AFM imaging and analysis were carried out using the Bruker MultiMode AFM with scanasyst, PeakForce QNM imaging modes, Peak Force Tapping technology, and NanoScope Analysis software.

### Statistical analysis

Unless otherwise indicated, all data are plotted as means ± S.D. Significant differences between groups were determined by two-tailed Student's *t*-test.

## Supporting information

S1 Text Supporting materials and methods. (DOCX)

S1 Fig DHX29 positively regulates intracellular (IC) HMW Poly(I:C)-induced type I IFN signaling. (**A-C**) Real-time PCR analysis of (**A**) *IFIT1*, (**B**) *IFIT2*, and (**C**) *CCL5* mRNA expression in Flag-*DHX29*- and empty vector-transfected 293T cells stimulated with IC HMW Poly(I:C). Data are plotted as the mean ± s.d. Results of (**A-C**) are representative of three independent experiments. *P < 0.05, **P < 0.01, ***P < 0.001 (two-tailed Student's t-test). Related to Fig 1 in the main text.
(TIF)

S2 Fig *DHX29* KD impairs MDA5-mediated type I IFN signaling. (**A**) Immunoblot analysis showing DHX29 expression in THP-1 cells stimulated with intracellular (IC) HMW Poly(I:C) (**A**, upper) or EMCV (**A**, lower) at the indicated time points. β-actin was used as a loading control. (**B**) Real-time PCR analysis of *IFNB* and *DHX29* mRNA levels in THP-1 cells stimulated with IC HMW Poly(I:C). (**C**) Real-time PCR analysis of the KD efficiency of *DHX29*-specific shRNAs. Scrambled (Scr) siRNA was used as a control. (**D**) 293T cells were cotransfected with HA-vector or HA*-DHX29* and Scr shRNA or *DHX29* shRNA. Cell viability was determined at various time points post-transfection using bromophenol blue. (**E**) ISRE-luciferase (Luc) (left panel) and IFN-β-Luc activities (right panel) in Scr shRNA- and *DHX29* shRNA-transfected 293T-TLR3 cells stimulated exogenously with naked Poly(I:C). (**F** and **G**) ISRE-Luc (left panel) and IFN-β-Luc (right panel) activities in Scr shRNA- and *DHX29* shRNA-transfected 293T cells stimulated with (**F**) IC LMW Poly(I:C) and (**G**) IC Poly(dA:dT). ISRE-Luc and IFN-β-Luc activities are expressed as the fold increase relative to the control. (**H-J**) Real-time PCR analysis of *IFIT1*, *IFIT2*, and *CCL5* mRNA levels in Scr shRNA- and *DHX29* shRNA-transfected 293T cells stimulated with IC HMW Poly(I:C). Data from (**A-J**) are plotted as the mean ± s.d. and are representative of three independent experiments. *P < 0.05, **P < 0.01, ***P < 0.001 (two-tailed Student's *t*-test). Related to Fig 2 in the main text.
(TIF)

S3 Fig *DHX29* KD reduces IC HMW Poly(I:C)-stimulated type I IFN signaling in human and mouse monocytes. (**A**) Real-time PCR analysis of the KD efficiency of *DHX29* siRNA in human PBMCs, human THP-1 cells, and mouse RAW cells. (**B, C**) Real-time PCR analysis of *IFIT2* and *CCL5* mRNA levels in scrambled (Scr) siRNA- and *DHX29* siRNA-transfected THP-1 cells stimulated with HMW Poly(I:C) or Poly(dA:dT). (**D, E**) Real-time PCR analysis of *IFIT2* and *CCL5* mRNA levels in Scr siRNA- and *DHX29* siRNA-transfected RAW cells stimulated with HMW Poly(I:C) or Poly(dA:dT) lyo/vec. Data from (**A-E**) are plotted as the mean ± s.d. and are representative of three independent experiments. *P < 0.05, **P < 0.01, ***P < 0.001 (two-tailed Student's *t*-test). Related to Fig 2 in the main text.
(TIF)

S4 Fig Western blot and Surveyor assay to evaluate the knockout efficiency of each sgRNAs and mixture virus. (**A, B**) Western blot of DHX29-sgRNA-Cas9 LentiCRISPR transduced 293T cells (**A**) or MEFs (**B**) after puromycin selection and SURVEYOR assays. (**C, D**) SURVEYOR assay of DHX29-sgRNA-Cas9 LentiCRISPR mixture transduced human PBMC (**C**) or murine cell (BMDC, pMAC) (**D**) after puromycin selection. (**E-G**) Immunoblot analysis of p-TBK1 in control sgRNA- or DHX29 sgRNA-transduced PBMC (**E**), BMDC (**F**), or peritoneal macrophages (pMAC) (**G**), left untreated or stimulated by LMW Poly(I:C). VSV, Poly(dA:dT), EMCV or HMW Poly(I:C). (**H**) Morphology of Control-Cas9 or DHX29-sgRNA-Cas9 LentiCRISPR transduced pMAC at Day 9 post transduction. Scale bar = 10μm. Related to Fig 3 in the main text.
(TIF)

S5 Fig DHX29 interacts with MDA5, but not LGP2 or RIG-I. (**A**) 293T cells were cotransfected with HA-mouse (m)*DHX29* and Flag-m*RIG-I*, Flag-m*MDA5*, or Flag-m*MAVS*. Whole cell lysates (WCL) were immunoprecipitated with anti-Flag beads and immunoblotted with anti-HA or anti-Flag antibodies. (**B**) Scrambled or *DHX29* siRNA transfected WT MEF and MAVS knockout MEF were stimulated with Flag-TBK1 overnight. The WCL were subjected to immunoblot with indicated antibodies. (**C**) Luciferase assay of RIG-I knockout 293T cell transfected with increase amount of DHX29, followed by stimulation of intracellular (IC) LMW Poly(I:C), HMW Poly(I:C), *MDA5* or *MAVS*. (**D**) RIG-I Knockout 293T cells were cotransfected with HA-*DHX29* and Flag-*MAVS* or Flag-*MDA5*. WCL were immunoprecipitated with anti-HA beads and immunoblotted with anti-HA or anti-Flag antibodies. Anti-RIG-I antibody is used to verify the RIG-I knockout cell in whole cell lysate. (**E**) HA-*DHX29*- and Flag-*MDA5* (20 ng)-transfected 293T cells were stimulated with intracellular (IC) HMW Poly(I:C) at the indicated time points. WCL were immunoprecipitated with anti-Flag beads and immunoblotted with anti-HA, phosphorylated (p)-IRF3, and IRF3 antibodies. (**F**) WCL obtained from THP-1 cells stimulated with IC HMW Poly(I:C) at the indicated time points were immunoprecipitated with anti-DHX29 antibody and immunoblotted with MDA5, p-IRF3, and IRF3 antibodies. (**G**) 293T cells transfected with HA-*DHX29* and Flag-*RIG-I* or Flag-*MDA5* were infected with indicated kind of stimulation at 8hr. The cell lysate was immunoprecipitated with anti-Flag beads and immunoblotted with anti-HA antibodies. (**H**) WCL obtained from 293T cells transfected with HA-*DHX29* and Flag-*LGP2* after 6hr EMCV treatment were immunoprecipitated with anti-Flag beads and immunoblotted with anti-HA and anti-Flag antibodies. (**I**) IFN-β- Luc activities in 293T cells transfected with indicated plasmids post HMW Poly(I:C) treatment were determined. (**J**) 293T cells expressing Flag-*MAVS* and Flag-*MDA5* were transfected with *DHX29* siRNA or scrambled (Scr) siRNA and then stimulated with IC HMW Poly(I:C). WCL were immunoprecipitated with anti-HA antibody and immunoblotted with anti-HA, anti-Flag, p-IRF3, and IRF3 antibodies. WCL were immunoprecipitated with anti-biotin beads and immunoblotted with anti-Flag and anti-HA antibodies. Data from (**C, I**) are plotted as the mean ± s.d. and are representative of three independent experiments. *P < 0.05, **P < 0.01, ***P < 0.001 (two-tailed Student's *t*-test). Related to Fig 5 in the main text.
(TIF)

S6 Fig DHX29 does not enhance the binding ability of MDA5 to LWM Poly(I:C) and mutations of DHX29 inhibit ISRE- luciferase activity in a concentration-dependent manner and do not affect phosphorylation level of RIG-I and MDA5. (**A**) WCL obtained from 293T cells co-transfected with Flag-tagged full-length (FL)-MDA5, N-MDA5, M-MDA5, or C-MDA5 and HA-DHX29a were immunoprecipitated with anti-Flag beads. The immunoprecipitated product was immunoblotted with anti-HA and anti-Flag antibodies. (**B**) 293T cells were cotransfected with ISRE-luciferase (Luc) and FL-DHX29, empty vector (vec), DHX29a, DHX29b, DHX29c, or DHX29d plasmids and stimulated by overexpressing MDA5. ISRE-Luc activity was normalized to the Renilla luciferase internal control and presented as the fold increase relative to stimulated FL-DHX29 control cells. (**C**) 293T cells transfected with Flag-*MAVS*, HA-*DHX29*, Flag-*MDA5*, or Flag-*MDA5* plus HA-*DHX29* were incubated with biotin-labeled LMW Poly(I:C) for 4 h. (**D**) ISRE-luciferase (Luc) activity in 293T cells transfected with *DHX29* and increasing concentrations (0, 100, and 200 ng per well) of WAM-*DHX29*, WBM-*DHX29*, *DHX29a*, or *DHX29b* and stimulated with intracellular (IC) HMW Poly(I:C). ISRE-Luc activity is expressed as the fold increase relative to the unstimulated control. (**E, F**) 20ng Flag-RIG-I (**E**) or Flag-MDA5 (**F**) transfected 293T cells were co-transfected with wildtype DHX29, DHX29c, WAM and WBM of DHX29, followed by stimulation for 6 hrs or not. The lysates were immunoprecipitated with anti-Flag beads and immunoblotted with indicated antibodies. Cal A was added 1hr before lysate collection. Data from (**B, D**) are plotted as the mean ± s.d. Results are representative of three independent experiments. *P < 0.05, **P < 0.01, ***P < 0.001 vs. IC Poly(I:C)-stimulated cells (two-tailed Student's *t*-test). Related to Fig 6 in the main text.
(TIF)
