# Supplementary material for: DHX29 functions as an RNA co-sensor for MDA5-mediated EMCV-specific antiviral immunity
Source: PLoS Pathog. 2018 Feb 20;14(2):e1006886. doi: 10.1371/journal.ppat.1006886 (PMC5834211; doi:10.1371/journal.ppat.1006886)
Supplement: S1 Text — (DOCX) [file ppat.1006886.s001.docx]

**DHX29 functions as a RNA co-sensor for MDA5-mediated EMCV-specific antiviral immunity**

**Supporting Materials and Methods**

**Reagents**

HMW Poly(I:C), LMW Poly(I:C), Poly(dA:dT), Poly(I:C), Poly(I:C)-HMW/lyovector, Poly(I:C)-LMW/lyovector, and Poly(dA:dT)/lyovector were purchased from Invivogen. *DHX29* and scrambled siRNA and shRNA plasmids were purchased from Life Technologies. The following antibodies were used: anti-total IRF3 (sc-9082) and anti-MDA5 (#sc-365630) from Santa Cruz Biotechnology; horseradish peroxidase–anti-Flag (M2) and anti-β-actin (#A1978) from Sigma; horseradish peroxidase–anti-HA (#11867423001) from Roche Applied Science; anti-phosphorylated TBK1, anti-phosphorylated IRF3, anti-RIG-I, anti-S8-p-RIG-I and anti-T170-p-RIG-I antibodies from Cell Signaling; anti-MAVS antibody from Enzo life; anti-S88-p-MDA5 is a gift from Michaela Gack lab (Harvard); anti-DHX29 from Santa Cruz (#sc-81080) and Cell signaling (#4648); Peripheral blood-derived mononuclear cell (PBMC) subsets were purified with Ficoll-Paque PLUS (GE Healthcare).Anti-Mouse CD4 PE (#12-0041-85), anti-mouse CD19 PE (#12-0193-82) and Anti-Mouse Ly-6G (Gr-1) PE (#12-9668-82) were purchased from eBioscience; EasySep Human Monocyte Enrichment Kit(#19059), EasySep Human Pan-Dendritic cell (DC) Pre-Enrichment Kit (#19251), EasySep PE Positive Selection Kit (#18557) were purchased from Stem cell Technologies; Dynabeads Untouched Human T Cells Kit (#11344D) and Dynabeads Untouched Human B Cells Kit (#11351D) were purchased from Thermo Fisher Scientific.

**Cells**

HEK293T (CRL-1573, human embryonic kidneys 293T), A549 (CCL-185, Adenocarcinomic human alveolar basal epithelial cells), NCI-H441 (HTB-174, human lung papillary adenocarcinoma cell line), MRC5 (CCL-171, human fetal lung fibroblast cell line), RAW264.7 (TIB-71, mouse Abelson murine leukemia virus transformed macrophage cell line) and Vero (African green monkey kidney cell line) cells were purchased from American Type Culture Collection (ATCC) and were maintained in high-glucose Dulbecco’s Modified Eagle’s Medium (Gibco) supplemented with 10% fetal bovine serum (FBS) and penicillin-streptomycin. THP-1 (Human acute monocytic leukemia cell line) cells were maintained in RPMI-1640 medium (Invitrogen) supplemented with 10% FBS and penicillin-streptomycin. Mouse Embryonic Fibroblasts (MEFs) were prepared from 15-day-old C57BL/6 embryos. *MDA5-/-*, MDA5 WT, *RIG-I-/-*, and RIG-I WT MEFs were kindly provided by Dr. S. Akira (Department of Host Defense, Osaka University, Japan). PBMCs were purified from the buffy coats of healthy donors (Gulf Coast Regional Blood Center, Houston, TX) by Lymphoprep (Nycomed Pharm) density-gradient centrifugation. The PBMC purification protocol was conducted according to the institutional guidelines and approved protocols of Houston Methodist Research Institute. Human T and B cell were isolated from human PBMC by using Dynabeads negative selection kit. Human monocyte and dendritic cells were isolated from PBMC by using EasySep enrichment kit. Mouse Bone-marrow derived dendritic cell (BMDC), bone-marrow derived macrophage (BMDM) were isolated from mouse bone marrow with 7 days culture in appropriate medium. Mouse CD4 T cells and CD19 B cells were isolated by using PE-anti-CD4 and PE-anti-CD19 positive-selection, respectively. Mouse peritoneal macrophage and neutrophil were isolated by 4% sterile thioglycollate injection.

**Transfection**

THP-1 cells and PBMCs were transfected with plasmids and/or siRNA using the Amaxa Cell Line Nucleofector Kit V and Amaxa T Cell Nucleofector Kit (Lonza) according to the manufacturer's instructions, respectively. MEFs were transfected with siRNA using Lipofectamine 2000 LTX (Invitrogen) for KD experiments, whereas ectopic expression of murine *DHX29* in MEFs was generated by lentiviral transduction of MEFs

**Point Mutation Constructs**

Point mutations were introduced into DHX29 using the Quick-change II XL Site-Directed Mutagenesis Kit (Stratagene) according to the manufacturer’s protocol.

**Expression Plasmid Constructions**

Human MDA5, MAVS, DHX29, and RIG-I were constructed using the Gateway Cloning System (Life Technologies). The following primers were used for murine genes:

mDHX29-attb2F:

5’-GGGGACAAGTTTGTACAAAAAAGCAGGCTTCggcggcaaaaataagaaacac aaggcgc; mDHX29-attb2R, 5’-GGGGACCACTTTGTACAAGAAAGCTGGGTCC TAtcaattattctctgtttttatcaat; mMDA5-attb2F, 5’-GGGGACAAGTTTGTA CAAAAAAGCAGGCTTCtcgattgtctgttctgcagaggacagct; mMDA5-attb2R, 5’-GGGGACCACTTTGTACAAGAAAGCTGGGTCCTActaatcttcatcactatacaag cag; mRIGF-xhoI-MDA5RFP-I-attb2F, 5’-GGGGACAAGTTTGTACAAAAAAGC AGGCTTCacagcggagcagcggcagaatctgcaag; mRIG-I-attb2R, 5’-GGGGA CCACTTTGTACAAGAAAGCTGGGTCCTAtcatacggacatttctgcaggatcg; mMAVS-attb2F, 5’-GGGGACAAGTTT GTACAAAAAAGCAGGCTTCacatttgctgag gacaagacctataagt; and mMAVS-attb2R, 5’-GGGGACCACTTTGTACAA GAAAGCTGGGTCCTAtcactgggccaggcgcctactacgg. Genes were cloned into pENTRY plasmids and then further cloned into different destination vectors using the Gateway Plasmid Construction Kit (Life Technologies) according to the manufacturer’s protocols. The following primers were used for cloning human genes into dsRed and eGFP plasmids: R-Bam1-MDA5-RFP+2, 5’-GggccGGATCCCGatcc tcatcactaaataaacagcatt; F-xhoI-MDA5RFP, 5’-gggccCTCGAGgccaaca tgtcgaatgggtattccacag acgaga; F-xhoI-MDA5GFP+1, 5’-gggccCTCGA GGtcgaatgggtattccacagacgaga; R-Bam1- MDA5GFP, 5’-gggccCC TAGGctaatcctcatcactaaataaacagc; F-SalI-DHX29RFP+1; 5’-gggccGTCG ACGgccaacatgggcggcaagaacaagaaacacaaggct; and R-BamHI-DHX29 RFP+2, 5’-gggccGGATCCCGgttattctctgttttt atcaattccgt.

**Cytokine-release Assay**

Human and mouse IFN-β were detected using an ELISA kit (PBL Biomedical Laboratories) according to the manufacturer's protocol.

**Immunoprecipitation and Immunoblot Analysis**

For immunoprecipitation experiments, purified proteins or whole cell extracts obtained 24 h post-transfection or ligand stimulation were lysed and shaken on ice for 15 min. Whole cell lysates were incubated overnight with the indicated antibodies or protein A/G beads (Pierce). For immunoprecipitation with anti-Flag or anti-HA, anti-Flag or anti-HA agarose gels (Sigma) were added to the cell extracts. After incubation, beads were washed 5 times with 1 ml low salt lysis buffer, lysed with 4xSDS loading buffer, and subjected to SDS-PAGE (Bio-Rad). Proteins were transferred onto nitrocellulose membranes (Bio-Rad). Membranes were blocked and incubated with specific antibodies. Immunoblots were developed using the LumiGlo Chemiluminescent Substrate System (KPL). The lysis buffer we used is low salt lysis buffer (50mM Tris, pH7.5; 150mM NaCl; 1% Triton-X; 5mM EDTA; 10% (v/v) glycerol with protease inhibitor mixture).

**Real-time Polymerase Chain Reaction (PCR) Analysis**

Total RNA was isolated from cells or tissues, and first-strand cDNA was generated from total RNA using oligo-dT primers and reverse transcriptase II (Invitrogen). Real-time PCR was performed using specific primers and the ABI Prism 7000 analyzer (Applied Biosystems) with SYBR GreenER qPCR Super Mix Universal (Invitrogen). Target gene expression values were normalized to *hGAPDH*. The following primers were used: hGAPDH forward primer, 5´ TCAAGAAGGTGGTGAAGCAG; hGAPDH reverse primer, 5´GAGGGGAG ATTCAGTGTGGT; hISG54 forward primer, 5´ GGAGGGAGAAAACTCCTTGGA; hISG54 reverse primer, 5´ GGCCAGTAGG TTGCACATTGT; hISG56 forward primer, 5´ TCAGGTCAAGGATAGTCTGGAG; hISG56 reverse primer, 5´ AGGTTGTGTATT CCCACACTGTA; hIFNB forward primer, 5´ CATTA CCTGAAGGCCAAGGA; hIFNB reverse primer, 5´ CAATTGTCCAGTCCCAGAGG; mIFNB forward primer, 5´ TCA CCTACAGGGCGGACTTC; mIFNB reverse primer, 5´ TCTCTGC TCGGACCACCATC; mISG54 forward primer,

5´ CAGCAAGATGCAACCAAGATG; mISG54 reverse primer,

5´ TCTCCAGTGACTCCTTACTC; mISG56 forward primer, 5´ TGCGATCCACAGTG AACAAC; mISG56 reverse primer, 5´ ACTTCCGGG AAATCGATGAG; mGAPDH forward primer, 5´ AGGTCGGTGTGAACGGATTTG; mGAPDH reverse primer, 5´ TGTA GACCATGTAGTTGAGGTCA; VSV-G forward primer, 5´ CAAGTCAAAATGCCCAAGA GTCACA; VSV G reverse primer, 5´ TTTCCTTGCA TTGTTCTACAGATGG; EMCV forward primer, 5´ TTGAAAGCCGGGGGTGGGAGATCC; and EMCV reverse primer, 5´ TCTGTTGTTATTTTGGGGTGGC; hDHX29 forward primer #1, 5′ GGGAGCTACTTTAGCCCTTTACC 3′ ; hDHX29 reverse primer #1 5′ CTCCAGCCAAACATCTCGGT 3′; hDHX29 forward primer #2, 5′ TCAGCACCTGGGAGCTACTT 3′ ; hDHX29 reverse primer #2 5′ TCTGCATCACTCCACTCCAG 3′.

**Surveyor Nuclease Assay for Genome Modification.**

293T and MEF cells were transduced with Control or various DHX29-sgRNA LentiCRISPR. The transduced cells underwent puromycin selection for 2 days (2 μg/ml for 293T cells and 6 μg/ml for MEF cells). Genomic DNA was extracted using the Quick-gDNA Kit (Zymol Research) according to the manufacturer’s protocol. The genomic region flanking the CRISPR target site for *DHX29* was PCR amplified: human *DHX29* forward: GAAGCCCGGATCGAAGCGCAGGGT; human *DHX29* reverse: CGTTAGCCTAGCTAGCTGAGATGGT; mouse *DHX29* forward: CGGGAGCCGAGAGGCGGAGTCTGA; and mouse *DHX29* reverse: AGATCTGACAGAACATGGAGCTGC. The PCR products were purified using the Gel DNA Recovery Kit (Zymol Research) according to the manufacturer’s instructions. Purified PCR products from control and test cells were mixed and subjected to a reannealing process to produce heteroduplex formation according to SURVEYOR Mutation Detection Kit (Transgenomic) protocol. After reannealing, products were treated with SURVEYOR nuclease and enhancer S (Transgenomic). The digested products were then subjected to electrophoresis on a 2% agarose gel with SYBR gold stain.
