# Supplementary figures and images for: DHX29 functions as an RNA co-sensor for MDA5-mediated EMCV-specific antiviral immunity
Source: PLoS Pathog. 2018 Feb 20;14(2):e1006886. doi: 10.1371/journal.ppat.1006886 (PMC5834211; doi:10.1371/journal.ppat.1006886)

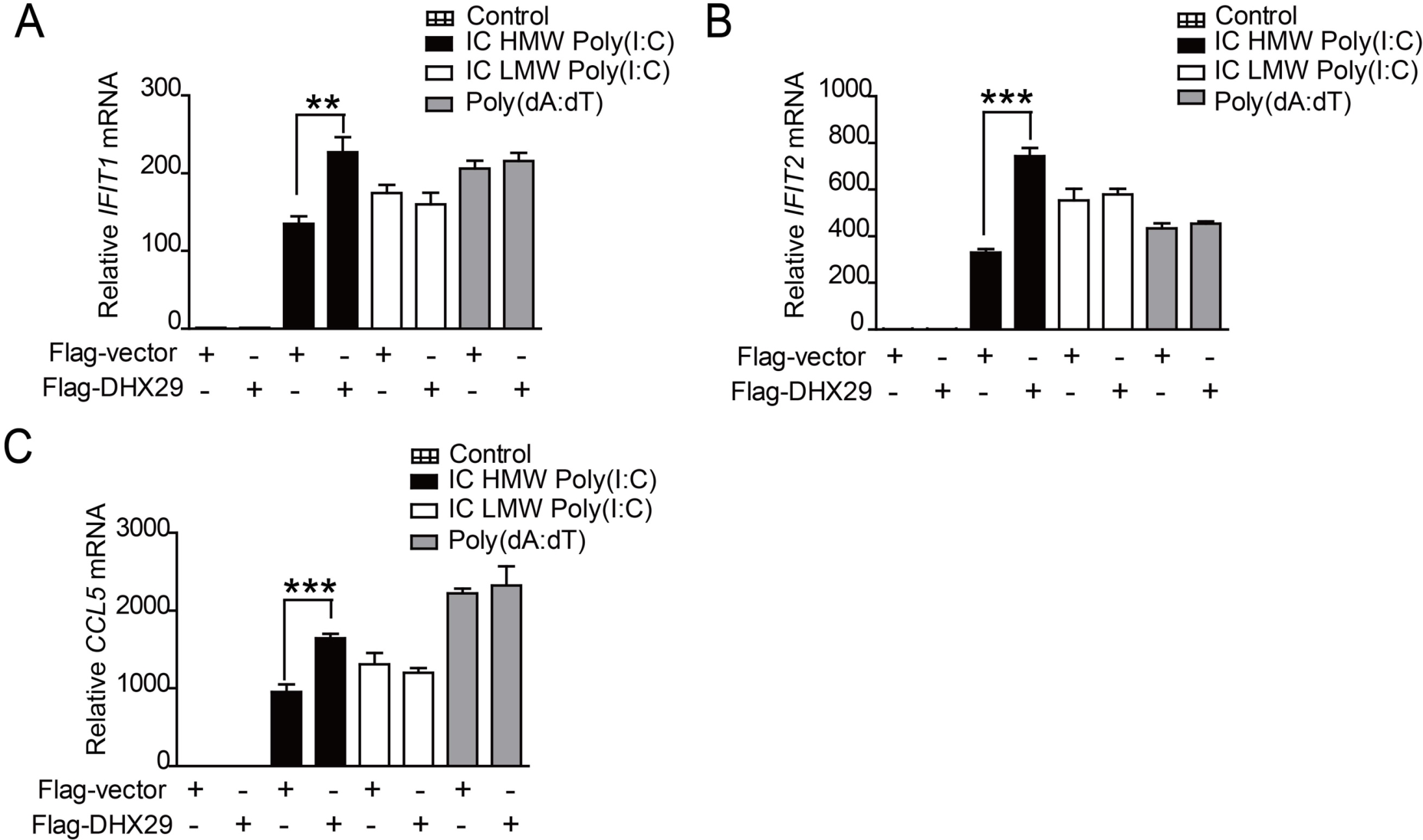

Supplement: S1 Fig — (A-C) Real-time PCR analysis of (A) IFIT1, (B) IFIT2, and (C) CCL5 mRNA expression in Flag-DHX29- and empty vector-transfected 293T cells stimulated with IC HMW Poly(I:C). Data are plotted as the mean ± s.d. Results of (A-C) are representative of three independent experiments. *P < 0.05, **P < 0.01, ***P < 0.001 (two-tailed Student's t-test). Related to Fig 1 in the main text. (TIF) [file ppat.1006886.s002.tif]

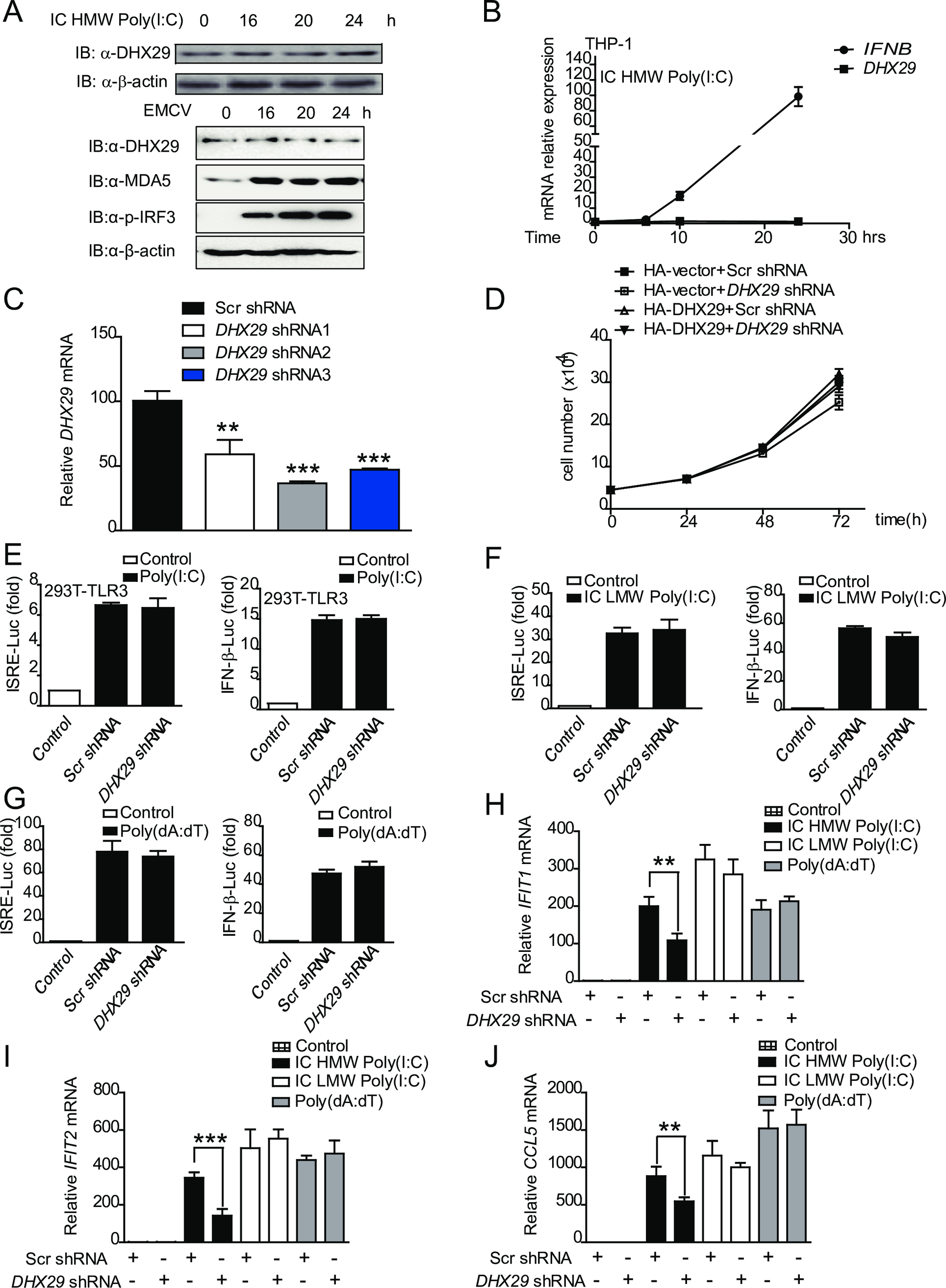

Supplement: S2 Fig — (A) Immunoblot analysis showing DHX29 expression in THP-1 cells stimulated with intracellular (IC) HMW Poly(I:C) (A, upper) or EMCV (A, lower) at the indicated time points. β-actin was used as a loading control. (B) Real-time PCR analysis of IFNB and DHX29 mRNA levels in THP-1 cells stimulated with IC HMW Poly(I:C). (C) Real-time PCR analysis of the KD efficiency of DHX29-specific shRNAs. Scrambled (Scr) siRNA was used as a control. (D) 293T cells were cotransfected with HA-vector or HA-DHX29 and Scr shRNA or DHX29 shRNA. Cell viability was determined at various time points post-transfection using bromophenol blue. (E) ISRE-luciferase (Luc) (left panel) and IFN-β-Luc activities (right panel) in Scr shRNA- and DHX29 shRNA-transfected 293T-TLR3 cells stimulated exogenously with naked Poly(I:C). (F and G) ISRE-Luc (left panel) and IFN-β-Luc (right panel) activities in Scr shRNA- and DHX29 shRNA-transfected 293T cells stimulated with (F) IC LMW Poly(I:C) and (G) IC Poly(dA:dT). ISRE-Luc and IFN-β-Luc activities are expressed as the fold increase relative to the control. (H-J) Real-time PCR analysis of IFIT1, IFIT2, and CCL5 mRNA levels in Scr shRNA- and DHX29 shRNA-transfected 293T cells stimulated with IC HMW Poly(I:C). Data from (A-J) are plotted as the mean ± s.d. and are representative of three independent experiments. *P < 0.05, **P < 0.01, ***P < 0.001 (two-tailed Student's t-test). Related to Fig 2 in the main text. (TIF) [file ppat.1006886.s003.tif]

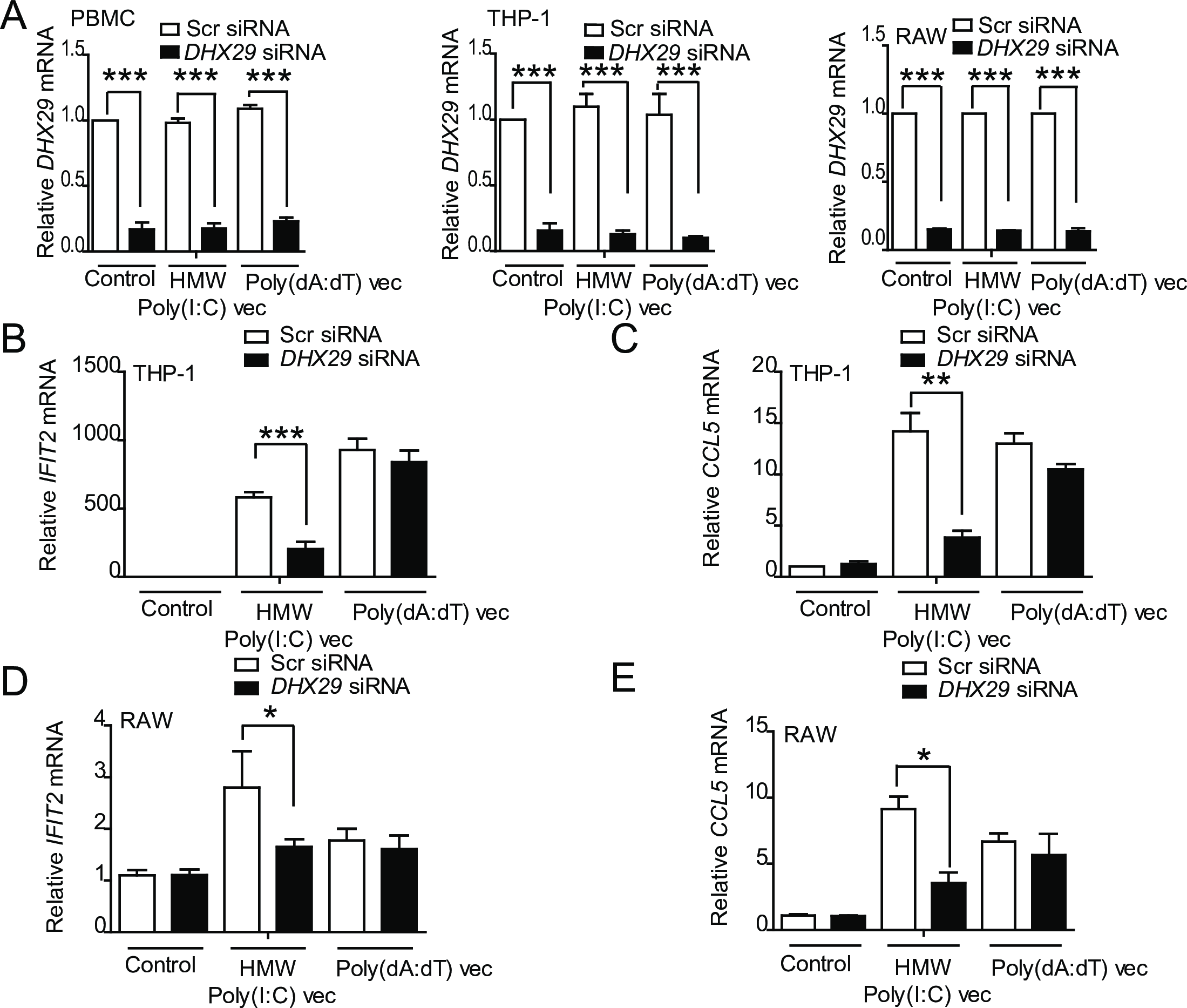

Supplement: S3 Fig — (A) Real-time PCR analysis of the KD efficiency of DHX29 siRNA in human PBMCs, human THP-1 cells, and mouse RAW cells. (B, C) Real-time PCR analysis of IFIT2 and CCL5 mRNA levels in scrambled (Scr) siRNA- and DHX29 siRNA-transfected THP-1 cells stimulated with HMW Poly(I:C) or Poly(dA:dT). (D, E) Real-time PCR analysis of IFIT2 and CCL5 mRNA levels in Scr siRNA- and DHX29 siRNA-transfected RAW cells stimulated with HMW Poly(I:C) or Poly(dA:dT) lyo/vec. Data from (A-E) are plotted as the mean ± s.d. and are representative of three independent experiments. *P < 0.05, **P < 0.01, ***P < 0.001 (two-tailed Student's t-test). Related to Fig 2 in the main text. (TIF) [file ppat.1006886.s004.tif]

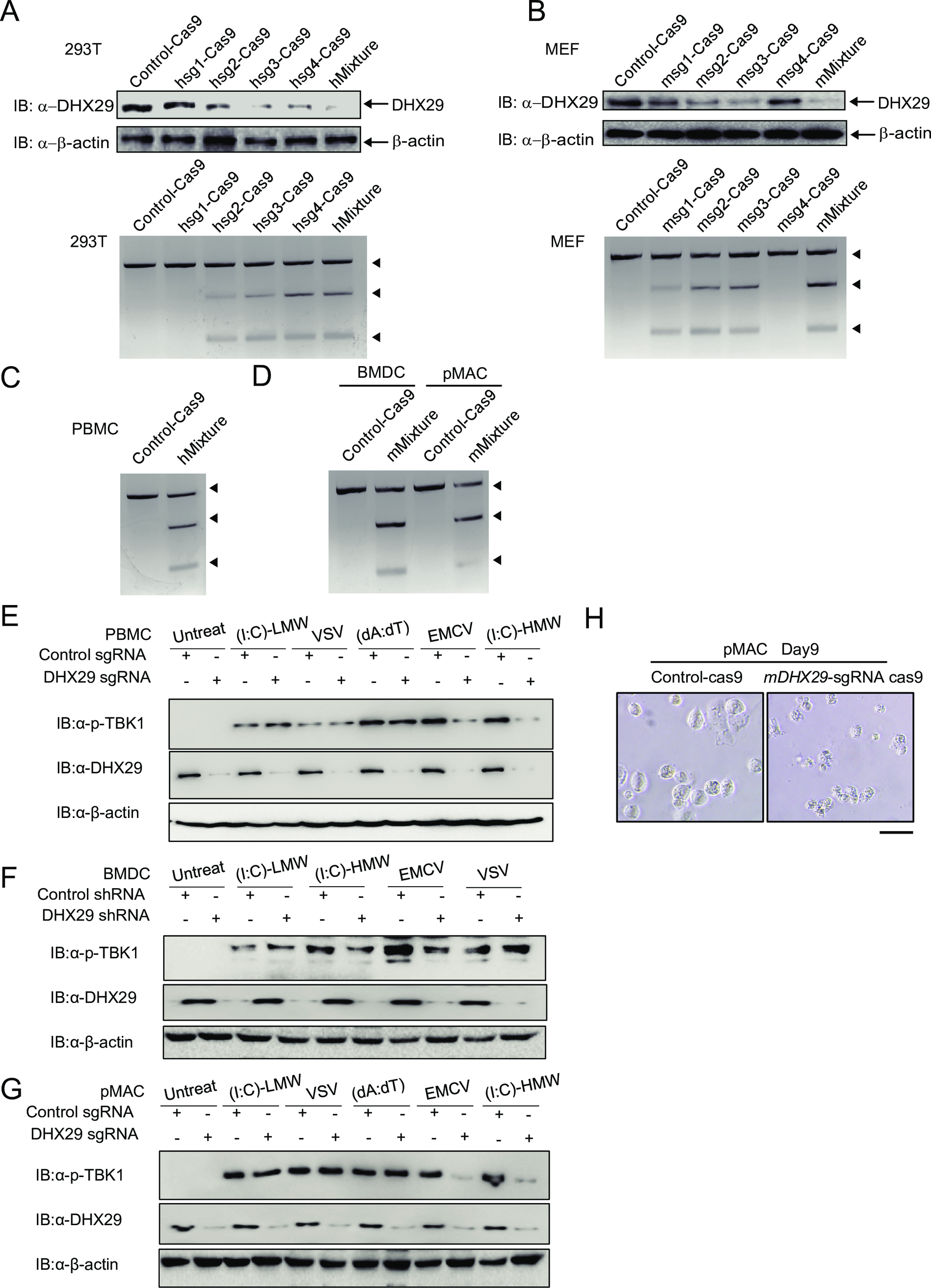

Supplement: S4 Fig — (A, B) Western blot of DHX29-sgRNA-Cas9 LentiCRISPR transduced 293T cells (A) or MEFs (B) after puromycin selection and SURVEYOR assays. (C, D) SURVEYOR assay of DHX29-sgRNA-Cas9 LentiCRISPR mixture transduced human PBMC (C) or murine cell (BMDC, pMAC) (D) after puromycin selection. (E-G) Immunoblot analysis of p-TBK1 in control sgRNA- or DHX29 sgRNA-transduced PBMC (E), BMDC (F), or peritoneal macrophages (pMAC) (G), left untreated or stimulated by LMW Poly(I:C). VSV, Poly(dA:dT), EMCV or HMW Poly(I:C). (H) Morphology of Control-Cas9 or DHX29-sgRNA-Cas9 LentiCRISPR transduced pMAC at Day 9 post transduction. Scale bar = 10μm. Related to Fig 3 in the main text. (TIF) [file ppat.1006886.s005.tif]

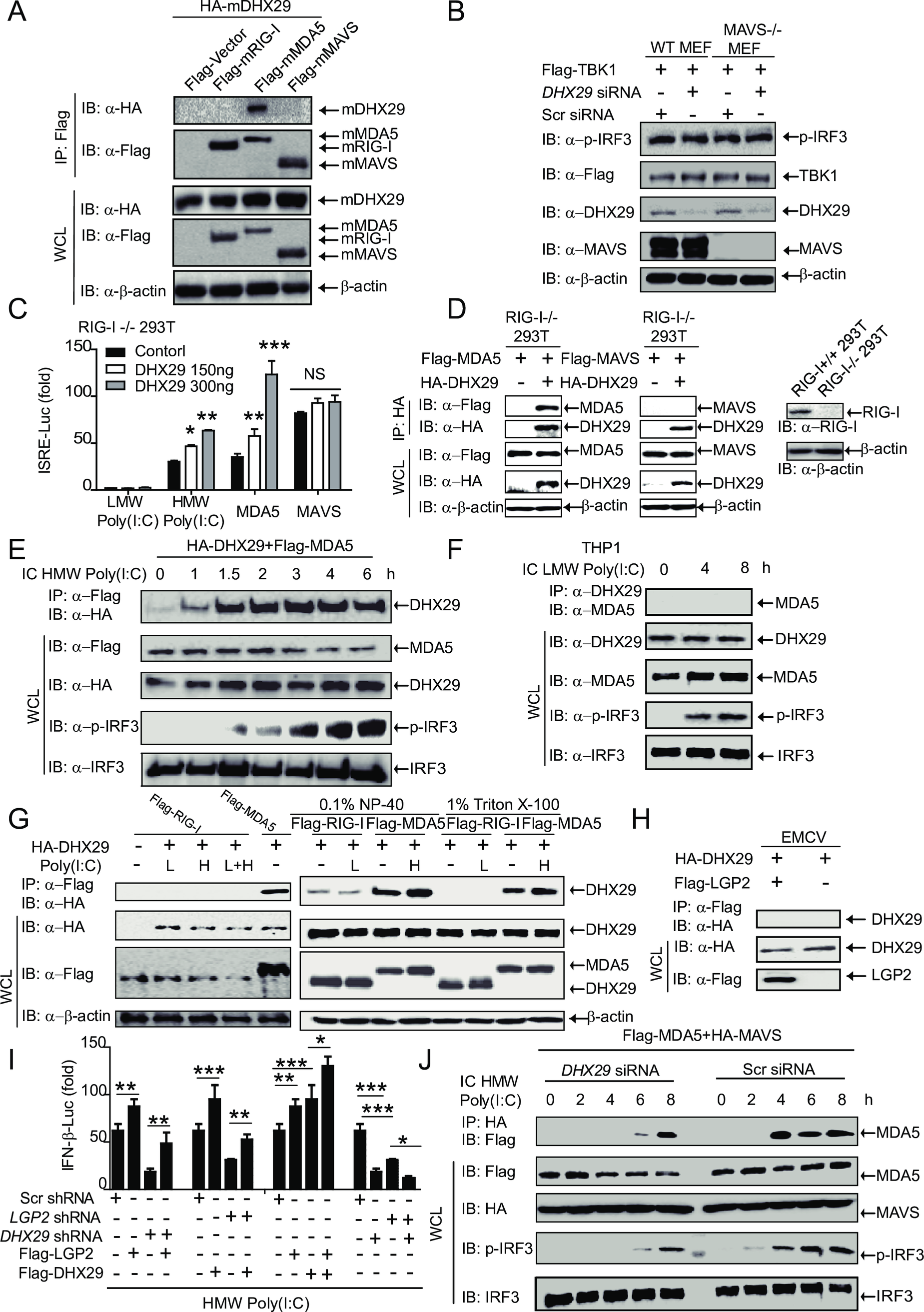

Supplement: S5 Fig — (A) 293T cells were cotransfected with HA-mouse (m)DHX29 and Flag-mRIG-I, Flag-mMDA5, or Flag-mMAVS. Whole cell lysates (WCL) were immunoprecipitated with anti-Flag beads and immunoblotted with anti-HA or anti-Flag antibodies. (B) Scrambled or DHX29 siRNA transfected WT MEF and MAVS knockout MEF were stimulated with Flag-TBK1 overnight. The WCL were subjected to immunoblot with indicated antibodies. (C) Luciferase assay of RIG-I knockout 293T cell transfected with increase amount of DHX29, followed by stimulation of intracellular (IC) LMW Poly(I:C), HMW Poly(I:C), MDA5 or MAVS. (D) RIG-I Knockout 293T cells were cotransfected with HA-DHX29 and Flag-MAVS or Flag-MDA5. WCL were immunoprecipitated with anti-HA beads and immunoblotted with anti-HA or anti-Flag antibodies. Anti-RIG-I antibody is used to verify the RIG-I knockout cell in whole cell lysate. (E) HA-DHX29- and Flag-MDA5 (20 ng)-transfected 293T cells were stimulated with intracellular (IC) HMW Poly(I:C) at the indicated time points. WCL were immunoprecipitated with anti-Flag beads and immunoblotted with anti-HA, phosphorylated (p)-IRF3, and IRF3 antibodies. (F) WCL obtained from THP-1 cells stimulated with IC HMW Poly(I:C) at the indicated time points were immunoprecipitated with anti-DHX29 antibody and immunoblotted with MDA5, p-IRF3, and IRF3 antibodies. (G) 293T cells transfected with HA-DHX29 and Flag-RIG-I or Flag-MDA5 were infected with indicated kind of stimulation at 8hr. The cell lysate was immunoprecipitated with anti-Flag beads and immunoblotted with anti-HA antibodies. (H) WCL obtained from 293T cells transfected with HA-DHX29 and Flag-LGP2 after 6hr EMCV treatment were immunoprecipitated with anti-Flag beads and immunoblotted with anti-HA and anti-Flag antibodies. (I) IFN-β- Luc activities in 293T cells transfected with indicated plasmids post HMW Poly(I:C) treatment were determined. (J) 293T cells expressing Flag-MAVS and Flag-MDA5 were transfected with DHX29 siRNA or scrambled (Scr) siRNA and [file ppat.1006886.s006.tif]

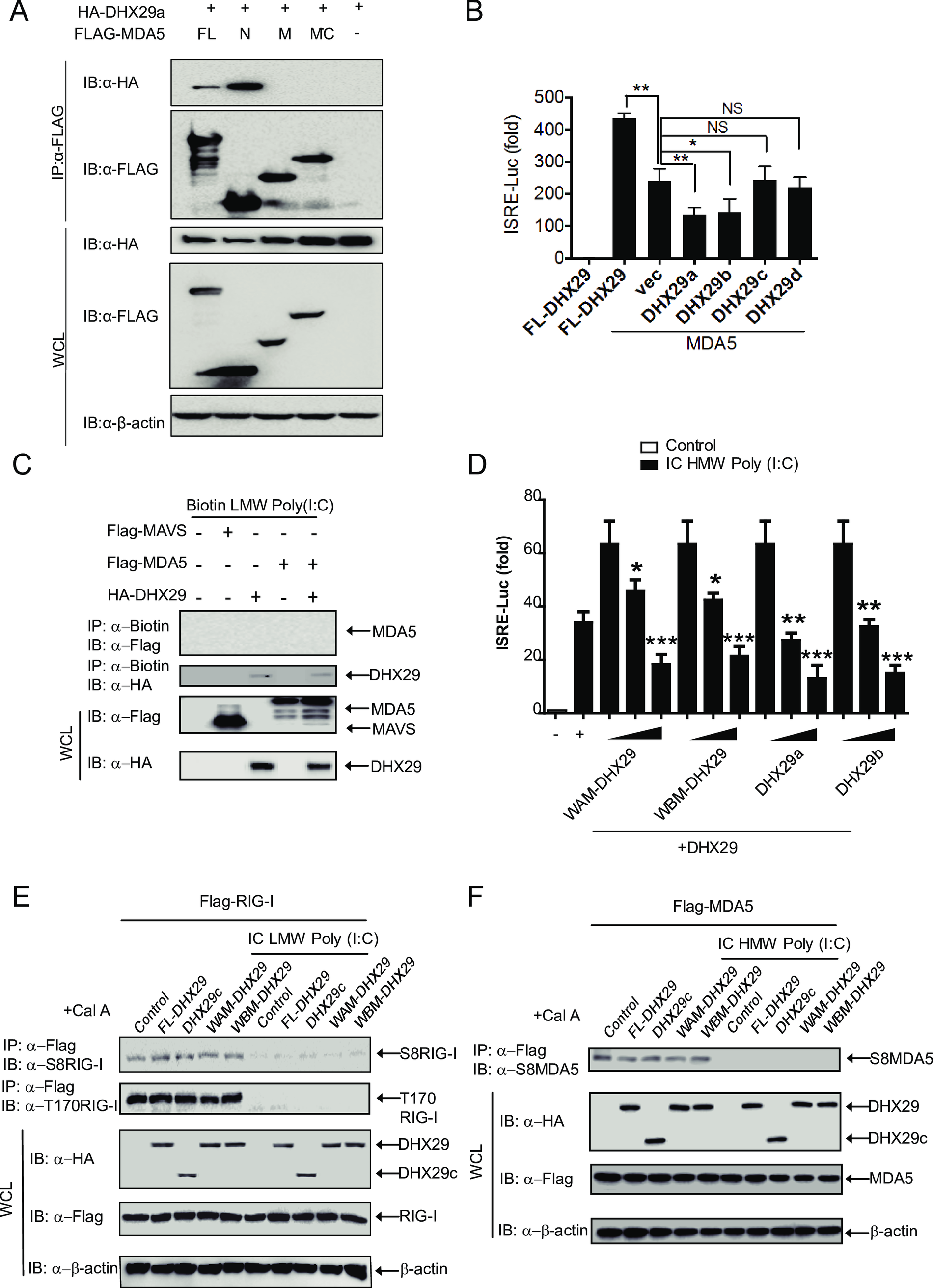

Supplement: S6 Fig — (A) WCL obtained from 293T cells co-transfected with Flag-tagged full-length (FL)-MDA5, N-MDA5, M-MDA5, or C-MDA5 and HA-DHX29a were immunoprecipitated with anti-Flag beads. The immunoprecipitated product was immunoblotted with anti-HA and anti-Flag antibodies. (B) 293T cells were cotransfected with ISRE-luciferase (Luc) and FL-DHX29, empty vector (vec), DHX29a, DHX29b, DHX29c, or DHX29d plasmids and stimulated by overexpressing MDA5. ISRE-Luc activity was normalized to the Renilla luciferase internal control and presented as the fold increase relative to stimulated FL-DHX29 control cells. (C) 293T cells transfected with Flag-MAVS, HA-DHX29, Flag-MDA5, or Flag-MDA5 plus HA-DHX29 were incubated with biotin-labeled LMW Poly(I:C) for 4 h. (D) ISRE-luciferase (Luc) activity in 293T cells transfected with DHX29 and increasing concentrations (0, 100, and 200 ng per well) of WAM-DHX29, WBM-DHX29, DHX29a, or DHX29b and stimulated with intracellular (IC) HMW Poly(I:C). ISRE-Luc activity is expressed as the fold increase relative to the unstimulated control. (E, F) 20ng Flag-RIG-I (E) or Flag-MDA5 (F) transfected 293T cells were co-transfected with wildtype DHX29, DHX29c, WAM and WBM of DHX29, followed by stimulation for 6 hrs or not. The lysates were immunoprecipitated with anti-Flag beads and immunoblotted with indicated antibodies. Cal A was added 1hr before lysate collection. Data from (B, D) are plotted as the mean ± s.d. Results are representative of three independent experiments. *P < 0.05, **P < 0.01, ***P < 0.001 vs. IC Poly(I:C)-stimulated cells (two-tailed Student's t-test). Related to Fig 6 in the main text. (TIF) [file ppat.1006886.s007.tif]
